# A developed DQ control method for shunt active power filter to improve power quality in transformers

**DOI:** 10.1371/journal.pone.0299635

**Published:** 2024-07-18

**Authors:** Saad F. Al-Gahtani, Z. M. S. Elbarbary, Shaik Mohammad Irshad

**Affiliations:** Electrical Engineering Department, College of Engineering, King Khalid University, Abha, Saudi Arabia; National Institute of Technology Silchar, India, INDIA

## Abstract

Power transformers are the most important component in power system. Exposing these transformers to the harmonic distortions causes additional heat losses, insulation stress, decrease in lifetime of insulation, and reduced power factor with decrease in efficiency of the system. The lifespan of distribution transformers is influenced by the fragility of power quality in power networks. Harmonic mitigation filters with robust control technique are required to reduce the harmonic effects on power transformer. Traditionally, synchronous reference frame (DQ) is employed to control the shunt active power filter (APF) for mitigation of harmonics in power transformers. DQ control of Shunt APF lacks merits of fast response, delayed operation due to phased lock loop under abnormal grid conditions leading to insufficient harmonic elimination. A developed DQ method based on detecting the positive and negative sequence components is proposed to precisely control the shunt APF for reliable operation of power transformer. This detection technique improves the response time, mitigate the harmonics effecting the operation of transformer and overall power factor. The proposed control system is evaluated under different abnormal operating scenarios and compared with traditional DQ method. The results and analysis confirm the efficacy of the developed DQ method in improving the power transformer performance.

## Introduction

Power quality refers to the characteristics of electrical power that affect the performance and reliability of electrical equipment and systems. It involves various parameters, including voltage, frequency, waveform shape, harmonics, transients, and interruptions. The purpose of power quality is to deliver electrical power to end-users within specified limits and without significant disturbances that can cause equipment malfunction or operational problems [1, 2]. Harmonic effect on the power system is one of the most frequently addressed issues for any power system. Some abnormal components are effective and dangerous, whereas others are insignificant [3]. The characteristics of the power supply have been affected by interruptions on the supply or load side. Inadequate power quality causes sensitive equipment to malfunction, leading to monetary, energy, and productivity losses [1, 2]. In a modern distribution system, power quality monitoring has become essential.

The rise of composite electrical equipment and the emerging use of solid-state nonlinear loads are the primary drivers of escalating power quality issues [4–6]. These elements lower system efficiency, lead to supply voltage distortion, and degrade the performance of other loads on the same distribution system.

### A. Research gap and motivation

Transformers are known as the core of a power system. Transformers are highly efficient and intended to handle linear loads at power frequency (50/60 Hz) [7, 8]. However, with the development of power electronic equipment, the number of non-linear loads has increased significantly. These devices frequently bring harmonic currents into the system and distorting the voltage waveform [9]. As a result, these devices would have a direct impact on the distribution transformer, which is designed to serve linear loads at a rated frequency. Temperature increases are expected as a result of increased core and winding losses caused by voltage and current harmonics [3, 7]. If the temperature at the winding hottest-spot rises over a typical value, the insulation may degrade, resulting in a reduced lifetime and an earlier transformer breakdown [3, 7, 10]. Several studies have demonstrated that harmonics can cause serious difficulties led to a variety of losses, including economic, human, and equipment life [2, 11, 12]. Consequently, research into harmonic effects has risen in order to develop acceptable solutions that can eliminate or lessen harmonic effects on transformers [13].

Concern has therefore increased over the severe effects of harmonics. The maximum permitted harmonic injection from the customer to the point of common coupling (PCC) and the overall harmonic distortion applied to the power system have been determined in accordance with IEC Standard 61000-3-6 [14] and IEEE Standard 519–2014 [15]. The capacity of distribution transformers is frequently derated to prolong the life of the transformers. The IEEE Standard C57.110–2008 has been created in order to de-rate distribution transformers having voltage ratings up to 69kV [15, 16].

### B. Contribution

In this article, the influence of current harmonics on power transformers is investigated through mathematical model and analysis. Then, mitigation techniques are investigated to identify the merits and demerits of each control technique. Among these techniques, a shunt APF has the ability to eliminate the power quality issues related to the currents [17–19]. To overcome the issues of shortage in performance of traditional DQ, a developed DQ method is implemented to control the shunt APF. A detection technique of positive and negative sequence components is integrated in the traditional DQ method. As a result of this integration, the developed DQ method avoids the use of the low-pass or high-pass filters which delay the control response. In addition, it enhances the performance of the phase-locked loop (PLL) in determining the phase angle of the grid voltage (θ) required for synchronization under abnormal grid conditions. This will allow adequate compensations of the currents from the shunt APF [20, 21]. The key features of are enhancing PLL functionality under abnormal grid conditions which allows fast and reliable synchronization with less computational effort of the control system, improving the elimination of current harmonics, providing nearly unity overall power factor, and maintaining almost the ideal transformation ratio of the transformer. Detailed Comparative performance analysis between the traditional and developed DQ methods is presented to demonstrate the superiority of the developed DQ control method.

### C. Organization of the article

The remaining paper is organized as follows: Literature review on harmonic effects on transformers, overall power factor’s relationship with displacement power factor, different harmonic mitigation techniques, and control of shunt APF are provided in Section II. Section III provides the configuration of proposed system, developed DQ control method. Section IV presents the results and discussion on the outcomes of the proposed system. Finally, Section V provides the conclusions of this paper.

## Literature review

### A. Harmonics effects on transformers

Identifying the sources of harmonics is required to evaluate their effects on transformers and other power system equipment [2, 12, 13]. Common harmonic sources include back-back diodes used in the public streetlamps, AC/DC converters, induction heating technology with power electronic switches, and passive power static compensation. In a practical and under rated operating conditions, a transformer normally introduces low distortion. However, if the voltage is deviated beyond the rating of the transformer, the ferro-magnetic core will be under saturation conditions leading to the high distortion in the voltage and current parameters of the circuit. Hence the transformer will slide into the non-linear mode of operation producing harmonics.

The difference between input and output power of a transformer is transformed into heat in core and winding. This heat is dissipated from the transformer’s exposed surfaces by a combination of convection and radiation [12, 22, 23]. As a result, both the core’s total surface area exposed and the windings affect how much heat is dissipated. Accurate forecasting of transformer temperature growth is difficult. One approach is to add the losses from the core and the winding assembly together and assume that the thermal energy is dissipated uniformly across the surface area of the assembly at all ambient temperatures. This is a reasonable assumption that the bulk of the transformer’s surface area is the ferrite core area rather than the winding area.

**Fig 1:**
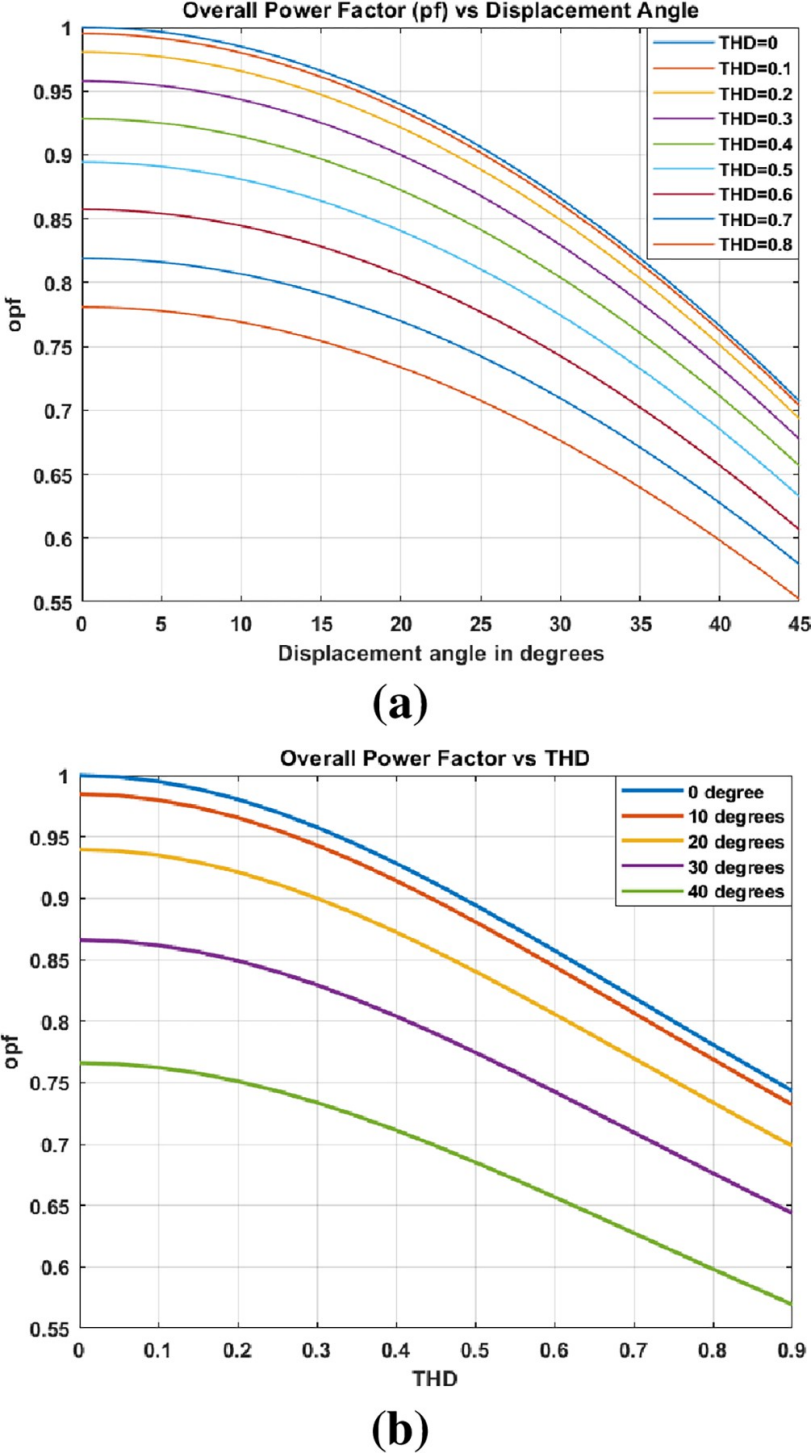
Effect on overall power factor Against (a) DPF and (b) THD.

To evaluate the harmonics effect on a transformer, the total harmonic distortion and transformer derating factor are essential [11].The total harmonic distortion (THD) indicates the amount of distortions in the voltage or the current and it can be represented as:

$${THD}_{X}=\frac{\sqrt{\sum_{h=2}^{\infty }{X_{h,rms}}^{2}}}{X_{1}}\times 100\%$$ (1)

where *X*_*h*,*rms*_ is the rms value of voltage or current harmonics, and *X*_1_ is the fundamental voltage or current. Both voltage and current harmonics affect transformer during no-load and load conditions [3, 11]. Eddy and hysteresis currents losses are the cause of no-load effect (core losses). Ohmic (copper losses), eddy current, and stray losses are the loading effect which leads to the increase of oil temperature [24]. Additional consequences could include reduced nominal power and transformer lifetime [25]. The transformer’s overall losses can be stated as follows [12, 13, 15, 26]:

$$P_{T=}P_{NL}+P_{LL}$$ (2)

where *P*_*T*_ is the total losses, *P*_*NL*_ is the no-load losses and *P*_*LL*_ is the load losses of the transformer. The losses that are caused by harmonics either on the core or on the winding result in increasing the heat in the winding because of increasing the values of the voltages and the currents much higher than the rated values [27, 28]. Consequently, the heat starts damaging the insulation of the transformers. If the heat continues, there will be no more insulation in the transformer. Thus, the transformer is no more functioning. Thermal stresses which is generated by nonlinear load are responsible for about 50% of transformer’s loss of life. In addition, the harmonics impact the power factor of the power system [29, 30]. As harmonics increase in the system, the overall power factor (*opf*) decreases as seen in the below equation [2]:

$$opf=\frac{1}{\sqrt{1+{THD}^{2}}}DPF$$ (3)

where *DPF* is the displacement power factor. The effect of DPF and THD on opf are depicted in Fig 1. All of these harmonic effects, in addition to transformer life, will have an impact on power transformer efficiency. Therefore, it is critical to reduce the amount of harmonics in transformers to perform efficiently and last longer.

### B. Mitigation techniques of harmonic effects on transformer

**Fig 2:**
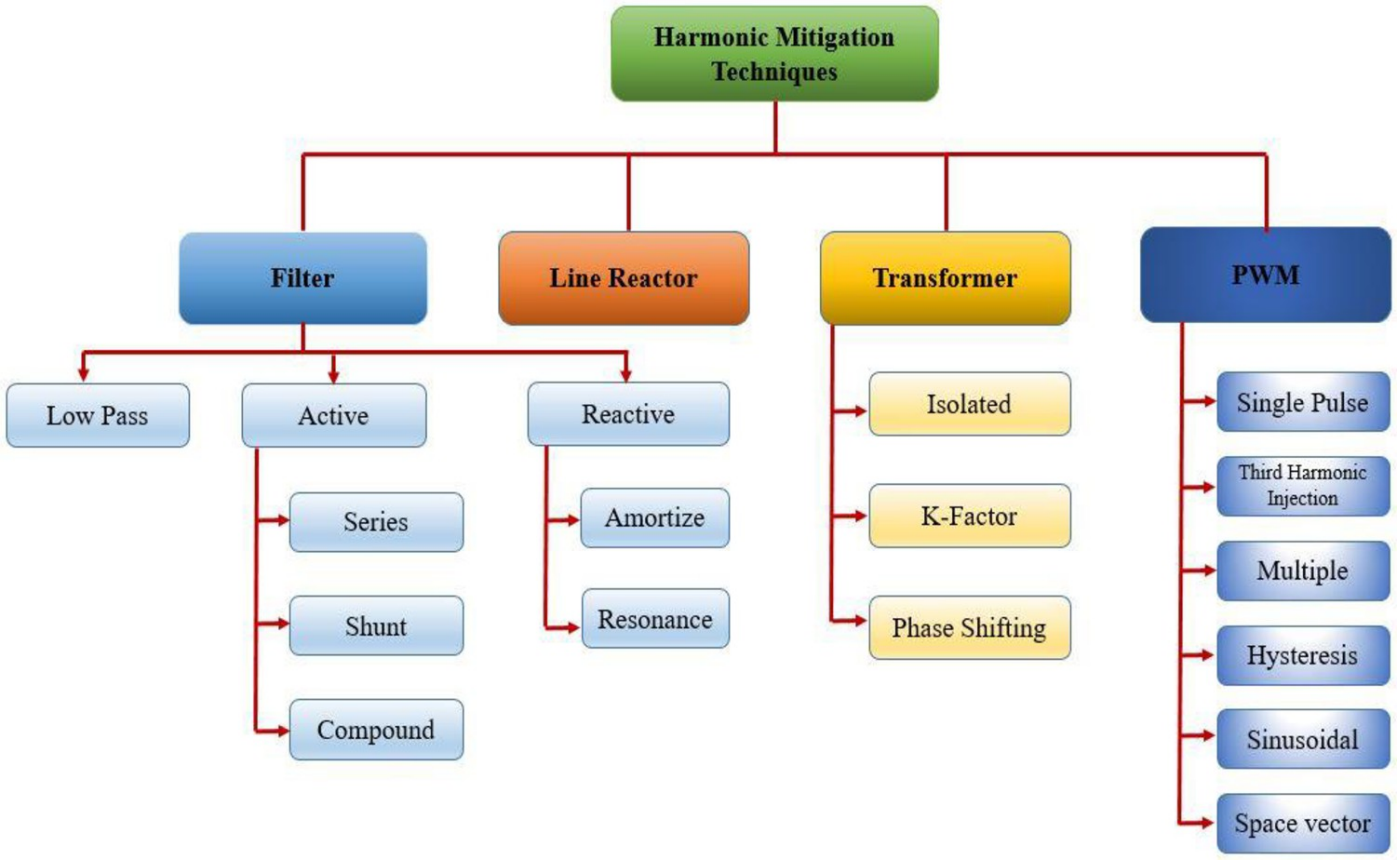
Harmonic mitigation techniques.

Numerous mitigation schemes for power systems are put forward to reduce the adverse impacts of harmonics [11, 31, 32]. Different types of mitigation techniques are listed in Fig 2. The merits and demerits of these mitigation techniques are presented in Table 1.

**Table 1:** Features of several harmonic mitigation techniques.

Type		Advantages	Disadvantages
**Filter**	**Passive/Low Pass**	•Cost-effective, low residual harmonics, •results can be predicted and guaranteed, •reduce cable heating and line loss [26, 38]	Must be connected to load in series, can be used only with nonlinear loads, increases the heating effect, lower life of linear loads, leads to low power factor [26, 38, 39]
	**Active**	Can mitigate up to 50^th^ order harmonics, can achieve less than 5% of distortion level [17, 40]	Complex power electronic circuit, requires more maintenance, high cost and more loss compared to passive filters [17, 40, 41]
**Line Reactor**	**AC and DC**	Reduce surge currents, Simple and reduce good level of harmonics at reasonable price [17, 26, 39]	Develops voltage drop, increase system losses, Not effective in completely mitigating harmonic distortions [26]
**Transformer**	**isolated**	Effective in both common and normal mode disturbance with good circuit isolation [26]	Size of the isolated transformer should match the rated load current, increase circuit losses, and High cost compared to line reactor [26]
	**K-Factor**	Ability to withstand the heat caused by eddy current losses, and the mixed (linear and nonlinear) load has a lower K-factor need than the nonlinear load [38].	Cost is high compared to an isolated transformer for each horsepower [26]
	**Phase Shifting**	5^th^ and 7^th^ order harmonics can be canceled at equal loading situation [17, 38, 41]	A bulky setup as two-phase shifting transformers are required, resulting in increase of losses [38, 39]
**Pulse Width Modulation**	**Single Pulse**	Simple to implement, easy to use and control [26]	Presence of harmonic content due to the single pulse, slow dynamic performance [26]
	**Multiple pulses**	Reliable and reduces THD less than 5% at loads [3, 26, 38].	High installation costs. Though harmonics are reduced, there is a reduction in efficiency. One setup for each product. Large footprint is required [26, 38, 41]
	**Sinusoidal**	Simple computation for reference generation, Improves the distortion factor, and eliminates higher harmonics when used with filters [26, 41].	Modulation (chopping) frequency needs to be synchronized with the inverter output frequency, which has the effect of over-modulation [38, 42].
	**Hysteresis**	• Ability to respond quickly to transients in load and line • easy to implement and robustness in load variation [22, 23]	Modulation frequency varies in a band for the fundamental frequency [22, 23]
	**Space Vector**	Can achieve low THD, low computational losses by preventing unnecessary switching, better modulation. Optimized efficiency and highly reliable [20, 43]	Reduction in volume and sometimes increase in switching losses [26]
	**Third Harmonic Injection**	Advantage of better utilization of the available DC. Inverter output voltage is raised by 15.5% without over modulation. Third harmonic can be completely eliminated [26]	Higher order harmonics still exists [26]

The development of APF allows simultaneous harmonic and reactive power compensation [33]. Regardless of whether the load is non-linear or in an unbalanced state, the APF has the capacity to maintain the primary current balance after mitigating [17, 34]. Due to the fact that most industrial applications need current harmonics correction, shunt active filters are more prevalent than series filters [18, 35]. By providing a compensation current, shunt APF seeks to reduce level of harmonics and reactive power components of the source current [18, 26, 36–38]. The performance of the shunt APF is evaluated by measuring the source currents THDs and amplitudes.

### C. Control of shunt APF

**Fig 3:**
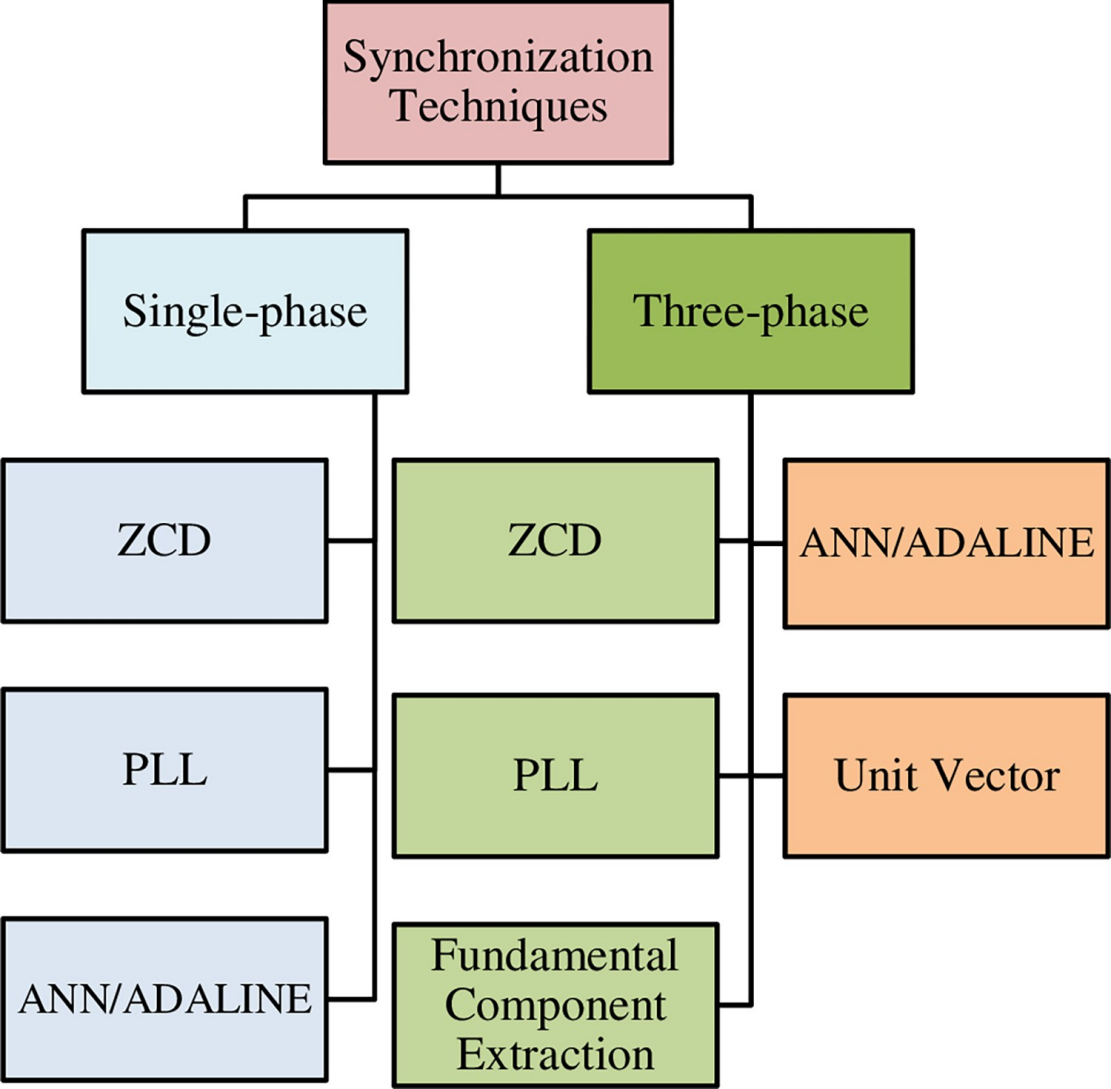
Synchronization techniques. To overcome these issues in traditional DQ, a modified DQ technique is proposed to control the shunt APF for minimizing the harmonics in the power transformers.

Control of shunt APF is influenced by the approaches used to generate command signals and controller, which controls switching pulse generation [17]. Several studies presented different control strategies [44, 45]. The effective two time-domain control methodologies are instantaneous reactive power (IRP) and synchronous reference (DQ) coordinate system approaches [39, 40, 42]. The extended IRP, the optimal-linear-prediction theorem, and the division frequency theorem are all dependent on modifying the IRP [46]. To extract the average or oscillating components of the real (P) and reactive (Q) power, some control systems use a low-pass filter (LPF) or a high-pass filter (HPF) [17, 39, 42]. Although LPFs and HPFs are simple to implement, but they induce phase delays at the fundamental frequency, which may compromise the shunt APF’s performance. For extracting fundamental components, the LPFs are replaced with a PLL. However, PLL performs poorly under the conditions of unbalanced or distorted grid voltages [17, 42]. Fig 3 presents different types of synchronization techniques [17, 26, 38].

## Proposed system

### A) System configuration

**Fig 4:**
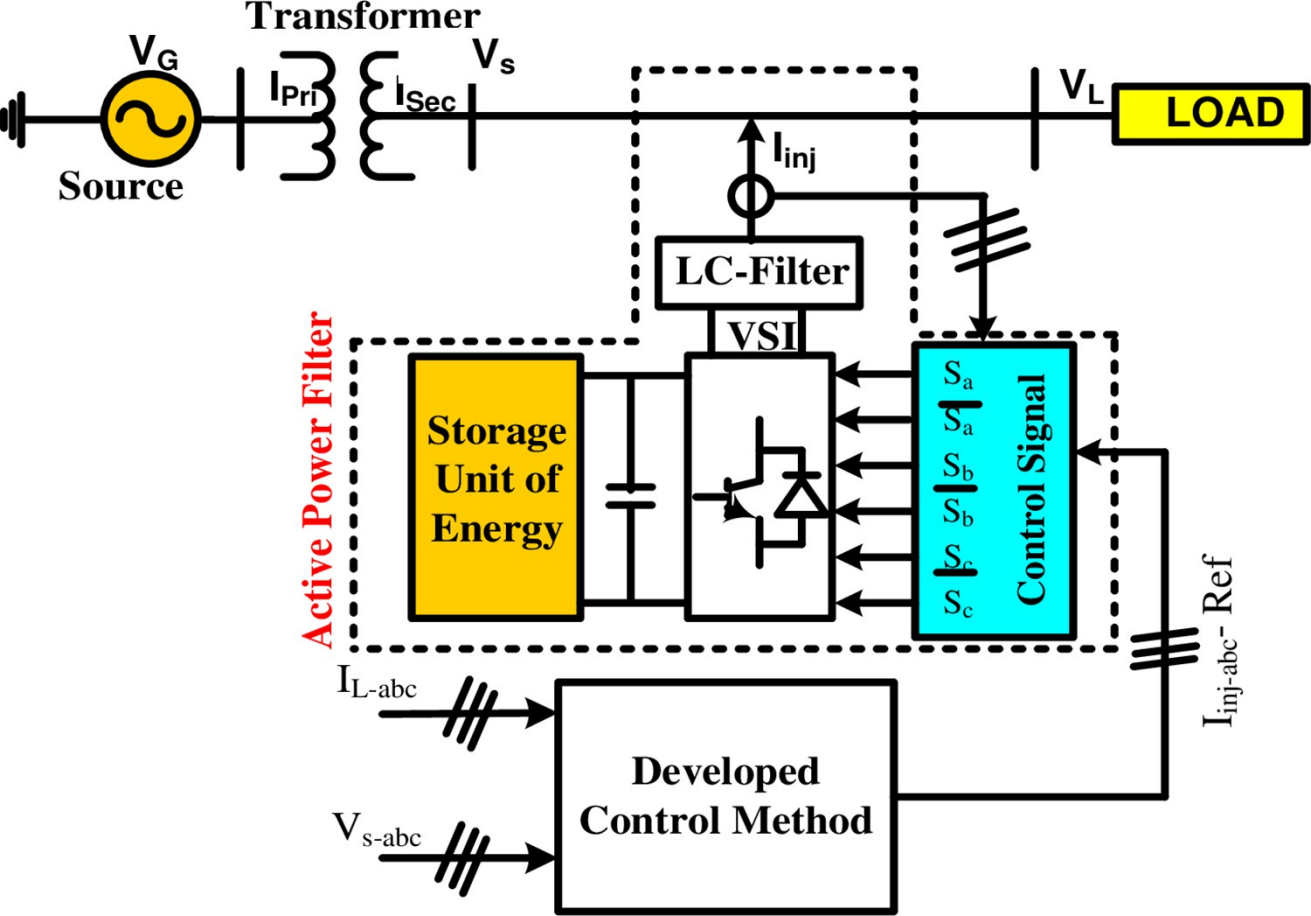
Block diagram of proposed control scheme with shunt APF control.

A power system is a complex circuit consisting of fundamental components including three-phase(3Φ) source connected to the nonlinear load (a resistive load supplied from 3Φ diode rectifier), through a 3Φ transformer of DYn11 vector group used commonly in distribution network. The nonlinear load reflects harmonic into the transformer increasing the losses. To mitigate the effect on loss level, a shunt APF circuit is inserted [47]. The shunt APF consists of a three-phase inverter, a DC power supply, and a three-phase LC switching-harmonic filter. The considered electrical system network diagram and parameters are shown in Fig 4 and Table 2.

**Table 2:** System parameters.

Details of Parameter		Value
**AC Source voltage**		13.8kV
**Line Frequency**		60Hz
**Transformer**	**Rated Power**	100kVA
	**Connection**	Dyn11
	**Rated Frequency**	50 or 60Hz
	**HV**	13.8kV
	**LV**	400V
	**Voltage Impedance**	4%
	**No Load Losses**	520W
	**Load Losses**	3200W
**Nonlinear Load**	10kW	
**LC filter**	2mH, C = 150μF, R_d_ = 0.2Ω	
**DC Voltage**	900V	

### B) Developed DQ control method

**Fig 5:**
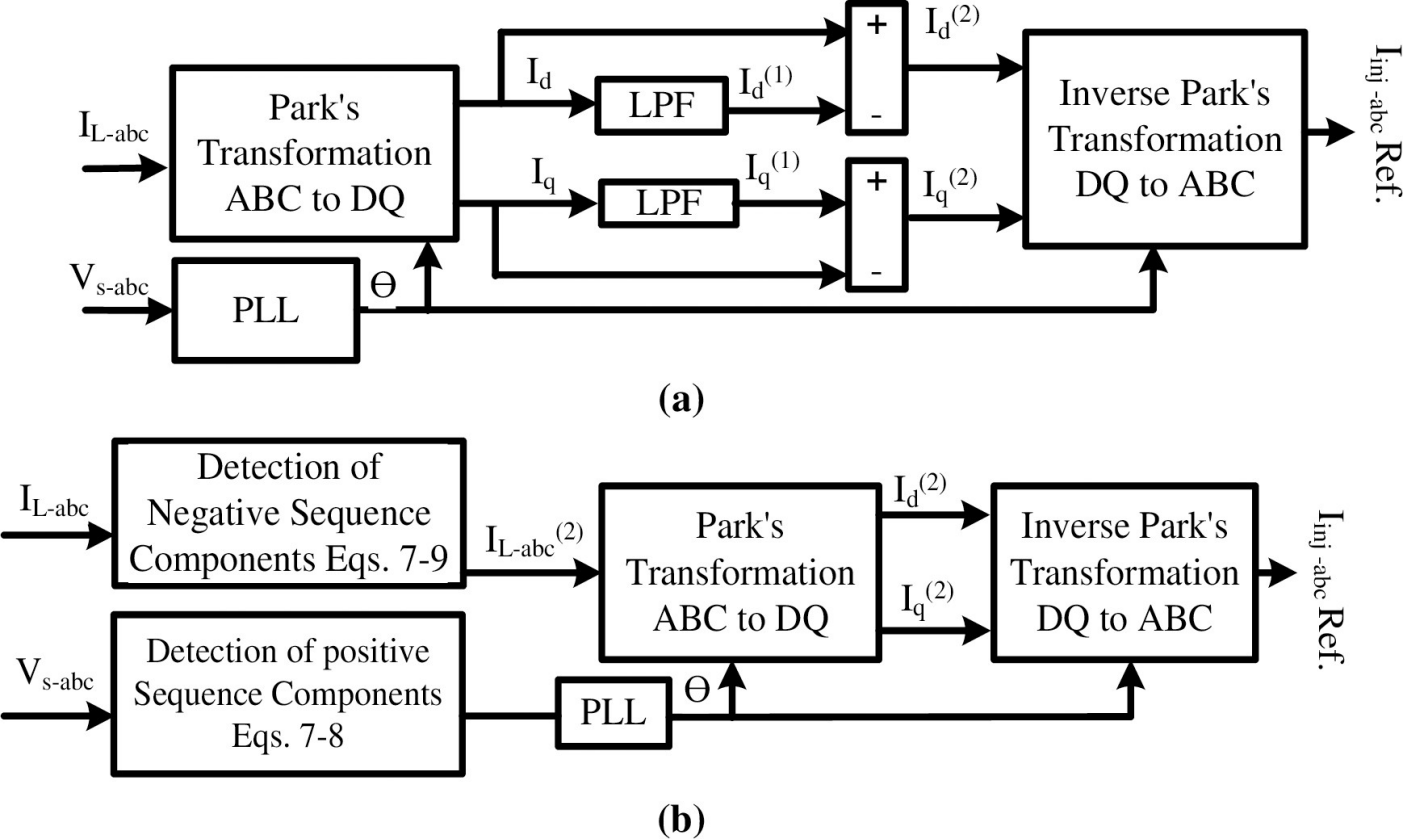
Block diagram of DQ control methods (a) the traditional, and (b) the developed.

The proposed control method of the shunt APF is based on modified DQ method as presented in Fig 5. To understand the control method, it is important to know that zero sequence currents and voltages are not present in delta-connected transformers since there is no neutral connection. Therefore, for systems with predominantly delta-connected loads and sources, the consideration of zero sequence components may be unnecessary. Also, system components with no grounding especially in industrial or isolated applications, grounding may be limited or absent. In such cases, zero sequence components may have minimal relevance, as they are primarily associated with ground faults and unbalanced ground currents. Considering this fact, the modified DQ method depends on appearing of the negative sequence components in load current during abnormal conditions. In the traditional DQ method, the PLL has a poor performance under severe harmonic distortion. Additionally, LPFs add delay to the injected signals. For that, the proposed control method combines the traditional DQ method and a detection technique of negative sequence components to enhance the performance of the PLL as well as to increase the elimination of harmonics.

#### (1) Detection technique

The detection of the negative sequence components is based on the transformation of symmetrical components [20]. This technique is performed in the time domain by converting the phasor rotation operators *a* and *a*^2^ to function of time. α and β are time-domain operator representing *a* and *a*^2^.


$$\alpha =1\left(t-\frac{240{}^{\circ}}{360{}^{\circ}\times f}\right)$$ (4)

$$\beta =1\left(t-\frac{120{}^{\circ}}{360{}^{\circ}\times f}\right)$$ (5)

Where f is the frequency with value 60Hz. Initially the positive sequence components of load currents are calculated and later negative sequence components are mathematically detected by subtracting the three-phase quantities from the positive sequence components. The transformation of the symmetrical components of load current is expressed as:

$$\left[\begin{array}{c} i^{\left(0\right)}\\ i^{\left(1\right)}\\ i^{\left(2\right)} \end{array}\right]=\frac{1}{3}\left[\begin{array}{ccc} 1 & 1 & 1\\ 1 & \alpha & \beta \\ 1 & \beta & \alpha \end{array}\right]\left[\begin{array}{c} i_{La}\\ i_{Lb}\\ i_{Lc} \end{array}\right]$$ (6)

Where i^(0)^, i^(1)^, and i^(2)^ are the zero, positive and negative sequence symmetrical components of load current.

The positive sequence component (i_L_(t)^(1)^) of the load currents in time domain is expressed as:

$${i_{L}(t)}^{\left(1\right)}=\frac{1}{3}(i_{La}(t)+\alpha i_{Lb}(t)+\beta i_{Lc}(t))$$ (7)

Then, the positive sequence components of each phase a, b, and c of the load current (i_L_(t)) is presented as:

$${i_{La}(t)}^{\left(1\right)}={i_{L}(t)}^{\left(1\right)},\;{i_{Lb}(t)}^{\left(1\right)}={\beta i_{L}(t)}^{\left(1\right)},\;{i_{Lc}(t)}^{\left(1\right)}=\alpha {i_{L}(t)}^{\left(1\right)}$$ (8)

Where i_La_(t)^(1)^, i_Lb_(t)^(1)^, and i_Lc_(t)^(1)^ are the positive sequence component of each phase a, b and c respectively in terms of positive sequence of load current (i_L_(t)^(1)^).

Now, the negative sequence components can be evaluated by the difference of load current and positive sequence component of load current in time domain system, and expressed as:

$$\left[\begin{array}{c} {i_{La}(t)}^{\left(2\right)}\\ {i_{Lb}(t)}^{\left(2\right)}\\ {i_{Lc}(t)}^{\left(2\right)} \end{array}\right]=\left[\begin{array}{c} i_{La}(t)\\ i_{Lb}(t)\\ i_{Lc}(t) \end{array}\right]-\left[\begin{array}{c} {i_{La}(t)}^{\left(1\right)}\\ {i_{Lb}(t)}^{\left(1\right)}\\ {i_{Lc}(t)}^{\left(1\right)} \end{array}\right]$$ (9)

Where i_La_(t)^(2)^, i_Lb_(t)^(2)^, and i_Lc_(t)^(2)^ are the negative sequence components of load current for phase a, b and c respectively.

### (2) Control method

The detection method is applied to measure the load currents. A PLL is used with the measured voltages on point of common coupling. The negative components of the load currents are converted into synchronous dq coordinates by implementing Park’s transformation:

$$\left[\begin{array}{c} i_{d}^{\left(2\right)}\\ i_{q}^{\left(2\right)} \end{array}\right]=\frac{2}{3}\left[\begin{array}{ccc} \cos\theta & \cos\left(\theta -120{}^{\circ}\right) & \cos\left(\theta +120{}^{\circ}\right)\\ -\sin\theta & -\sin\left(\theta -120{}^{\circ}\right) & -\sin\left(\theta +120{}^{\circ}\right) \end{array}\right]\left[\begin{array}{c} {i_{La}(t)}^{\left(2\right)}\\ {i_{Lb}(t)}^{\left(2\right)}\\ {i_{Lc}(t)}^{\left(2\right)} \end{array}\right]$$ (10)

Where $i_{d}^{\left(2\right)}$ and $i_{q}^{\left(2\right)}$ are the direct and quadrature components of the load current.

where *θ* = *ωt* is angular position determined by the PLL. The inverse Park’s transformation is then used to transform the negative sequence components of the shunt APF into the abc coordinate’s frame of reference:

$$\left[\begin{array}{c} i_{inja,ref}\\ i_{injb,ref}\\ i_{injc,ref} \end{array}\right]=\left[\begin{array}{cc} \cos\left(\omega t+\theta \right) & -\sin\left(\omega t+\theta \right)\\ \cos\left(\omega t+\theta -\frac{2\pi }{3}\right) & -\sin\left(\omega t+\theta -\frac{2\pi }{3}\right)\\ \cos\left(\omega t+\theta +\frac{2\pi }{3}\right) & -\sin\left(\omega t+\theta +\frac{2\pi }{3}\right) \end{array}\right]\left[\begin{array}{c} i_{d}^{\left(2\right)}\\ i_{q}^{\left(2\right)} \end{array}\right]$$ (11)

**Fig 6:**
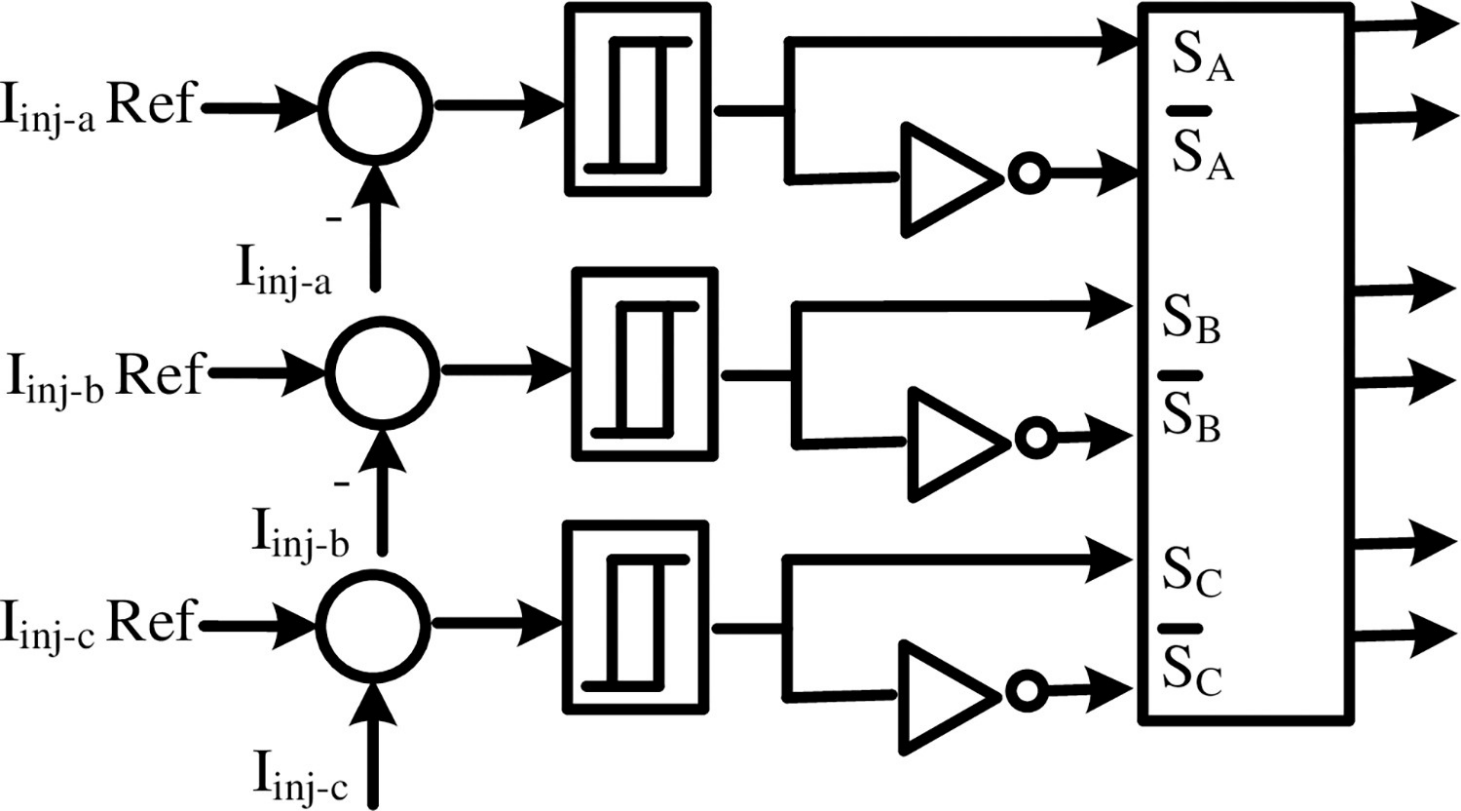
Hysteresis current controller.

The measured current from the shunt APF is compared to the reference current. A hysteresis current control is used to control and generate the firing signals for IGBT switches of the inverter switches as depicted in Fig 6.

## Results and discussion

**Fig 7:**
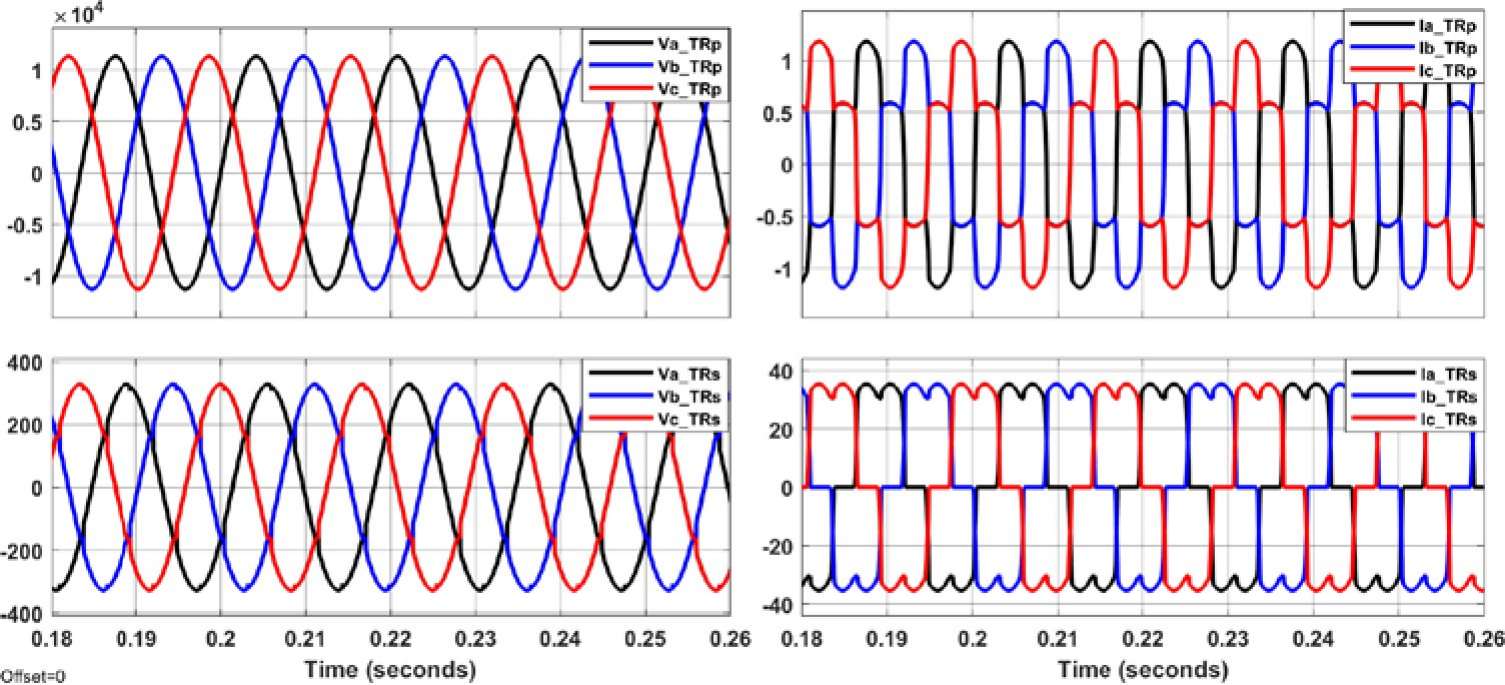
Primary and secondary voltages and currents with nonlinear load of 10kW and without the shunt APF.

**Fig 8:**
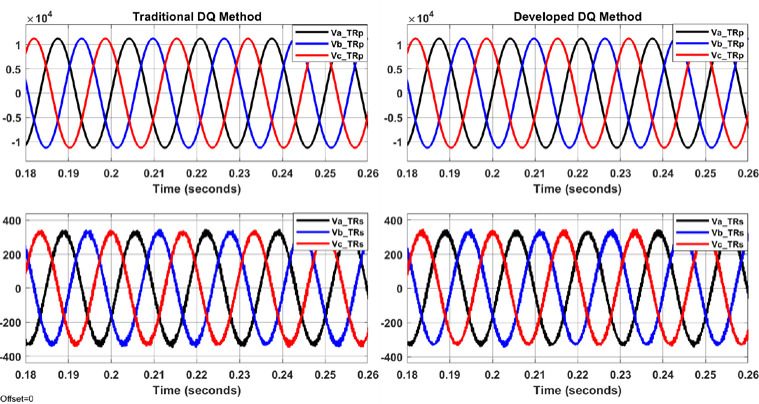
Transformer primary and secondary voltages with the shunt APF controlled by traditional DQ method (on the left) and by developed DQ method (on the right).

**Fig 9:**
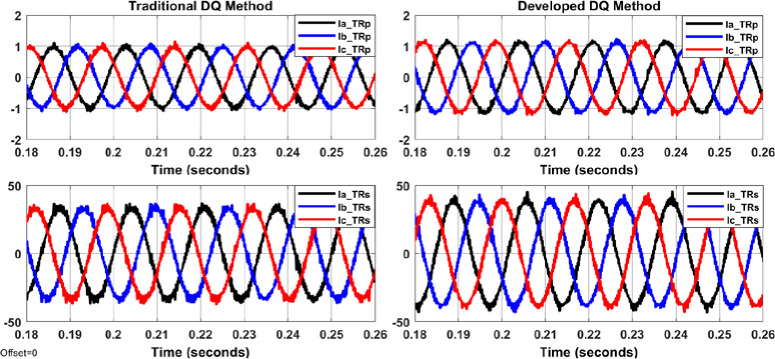
Transformer primary and secondary currents with the shunt APF controlled by traditional DQ method (on the left) and by developed DQ method (on the right).

**Fig 10:**
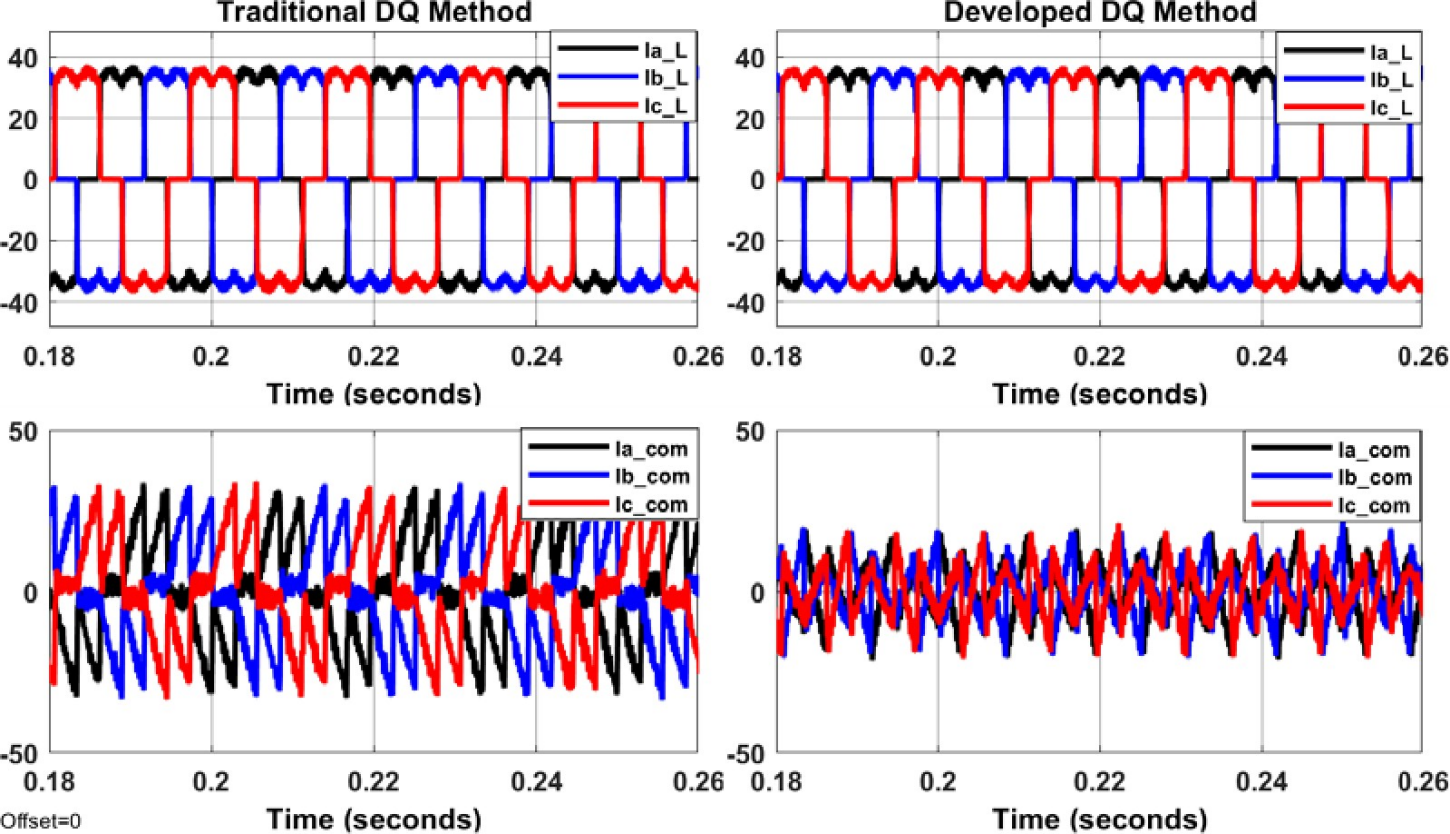
Load currents and compensated currents from the shunt APF controlled by traditional DQ method (on the left) and by developed DQ method (on the right).

**Fig 11:**
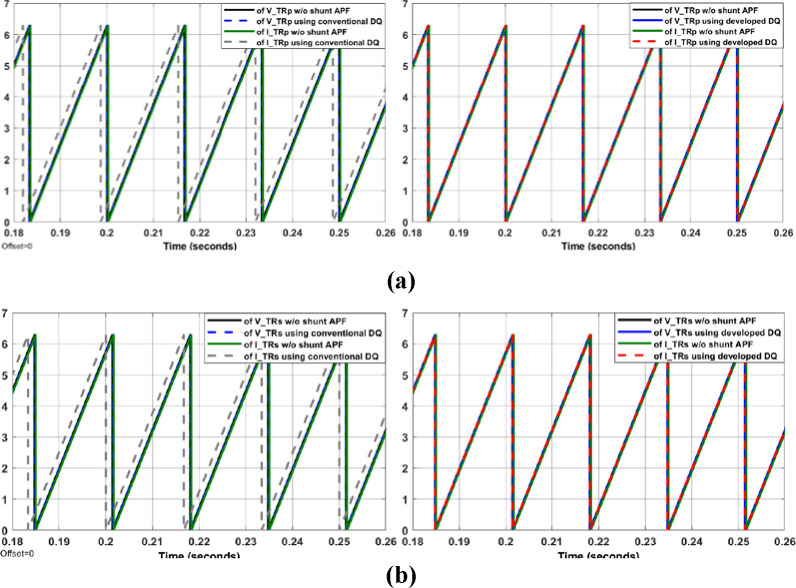
Tracking the phase angle of (a) primary voltage and (b) secondary voltage.

**Fig 12:**
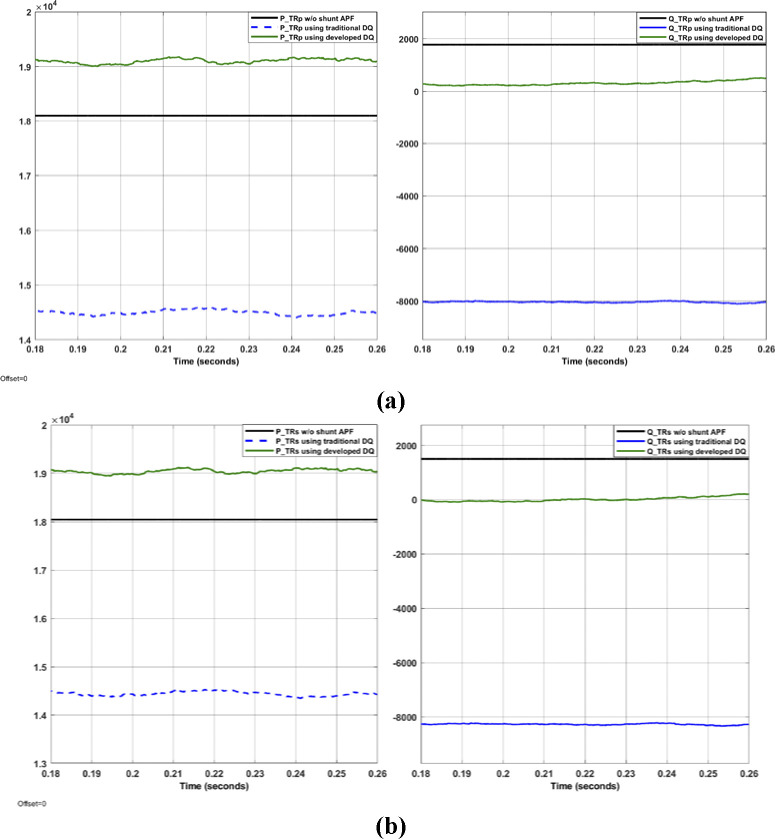
Power of (a) primary side and (b) secondary side.

The system shown in Figs 4–6 with aid of Eqs (1–11) are simulated to evaluate the control system response with and without the proposed APF using MATLAB/Simulink. Figs 7–12 show waveforms of the system variables with and without shunt APF. In addition, analysis of the system are provided in Tables 3–5. The analysis are obtained using Simulink steady state and FFT Analysis.

**Table 3:** Magnitudes and THDs of transformer voltage and current.

Parameter		Without Shunt APF	Conventional DQ	Developed DQ
**V**_**abc_TR_1**_	**V**_**peak**_	11267.65, 11267.65, 11267.65	11267.65, 11267.65, 11267.65	11267.65, 11267.65, 11267.65
	**THD**	0.01, 0.01, 0.01	0.01, 0.01, 0.01	0.01, 0.01, 0.01
**V**_**abc_TR_2**_	**V**_**peak**_	326.15, 326.15, 326.15	328.3, 328.3, 328.3	326.5, 326.5, 326.5
	**THD**	3.2, 3.2, 3.2	1.5, 1.5, 1.5	1.5, 1.7, 1.6
**I**_**abcTR_2**_	**A**_**peak**_	1.08, 1.08, 1.08	0.98, 0.98, 0.98	1.13, 1.13, 1.13
	**THD**	28, 28, 28	5.1, 5.15, 5.2	4.3, 4.4, 3.7
**I**_**abc_TR_2**_	**A**_**peak**_	37, 37, 37	33.85, 33.8, 33.86	38.98, 38.97, 38.95
	**THD**	28.13, 28.13, 28.13	5.14, 5.1, 5.2	3.9, 4, 4

**Table 4:** Efficiency and overall power factor of the system.

Parameters		θ_a_ (°)	θ_b_ (°)	θ_c_ (°)	P_primary_ and P_secondary_ (kW)	η (%)	PF
**Without Shunt APF**	**V**_**abc_TR_1**_	0	240	120	18.17	99.2	0.96
	**I**_**abcTR_1**_	-5.6	234.4	114.4			
	**V**_**abc_TR_2**_	-30.7	209.3	89.3	18.04		0.96
	**I**_**abc_TR_2**_	-35.4	204.6	84.6			
**Conventional DQ**	**V**_**abc_TR_1**_	0	240	120	14.5	99.6	0.88
	**I**_**abcTR_1**_	28.8	268.9	148.9			
	**V**_**abc_TR_2**_	-30.8	209.2	89.2	14.45		0.87
	**I**_**abc_TR_2**_	-0.9	239.1	119.2			
**Developed DQ**	**V**_**abc_TR_1**_	0	240	120	19.09	99.9	0.99
	**I**_**abcTR_1**_	-1.3	238.7	118.8			
	**V**_**abc_TR_2**_	-30.9	209.1	89.1	19.08		0.99
	**I**_**abc_TR_2**_	-31.1	208.9	88.9			

**Table 5:** Transformer ratio.

Condition of Transformer	Ratio
Ideal Transformer	34.50:1
Without shunt APF	34.55:1
Shunt APF controlled by traditional DQ method	34.30:1
Shunt APF controlled by developed DQ method	34.51:1

As displayed in Figs 7–10, the voltages and currents at the primary and secondary sides of the transformer with and without shunt APF. The currents are balanced and sinusoidal with the shunt APF. However, the voltage and current with the proposed control method have significant improvement compared to that of traditional method in terms of the THD and phase displacement between the voltage and current as shown in Table 3. Without the shunt APF, the current THD is about 28% at both sides of the transformer. This amount of THD has considerably decreased to about 5% under traditional control method and about 4% under the developed control method meeting the acceptable levels as per IEEE standards. Moreover, a proper synchronization between the phase voltage and phase current at 1 and 2 windings of the transformer is achieved using the modified DQ control method. The waveforms and values of the corresponding voltage and currents are presented in Fig 11 and Table 4; The synchronization and THD values from Tables 3 and 4 has shown the effectiveness of the developed control method in improving the overall power factor. As a result, the use of modified control method has increased the capacity transferred active power and has decreased the wasted or lost active power as depicted in Fig 12 and Table 4. The power efficiency of the transformer under the developed control method is also the highest among other systems.

Additionally, transformation ratio of the transformer is evaluated as shown in Table 5 where ideal transformation ratio is 34.5:1. Without the shunt APF, the ratio is 34.55:1 which indicates that the secondary voltage is reduced and secondary current is increased. This increase in secondary current leads to an increase in losses in winding leading to the rise in temperature of the secondary winding. This temperature increase effect the life of the transformer. With the shunt APF controlled by the traditional method, the ratio is 34.3:1 meaning secondary voltage is increased effecting the secondary winding insulation. Finally, the ratio with the developed DQ control is 34.51:1 which means the transformer is employed under ideal rated state of the transformer. Due to this the transformer will have the capability to withstand the loss and temperature effect.

## Conclusion

Harmonics are undesirable quantities that affect power transformers’ performance. No-load and load losses influenced by harmonics increase the heat of the transformer which consequently weakens the insulation of the transformer and reduces its lifespan. Therefore, eliminating the current harmonics and power quality issues before reaching the transformers is must.

Shunt APFs can provide a great solution for eliminating power quality issues associated with system currents. The study has introduced a developed DQ control method for shunt APF. The method contains an efficient technique by eliminating LPFs and implementing the detection of positive and negative sequence components. The technique improves the response and reliability of PLL synchronization and the capability of the shunt APF in removing the current harmonics.

A comparative study between the traditional DQ control and the developed control were provided. The results has validated the superiority of the developed DQ approach in minimizing reactive and harmonic components, synchronizing the phase angles between the voltage and currents, providing fast response, improving the transformer’s transformation ratio and efficiency, better capacity of transferred active power, and maintaining almost unity power factor. The proposed new DQ control method for shunt APF provides a protection wall from harmonic risks that threaten the performances and life span of power transformers. This research work can be extended by using evolutionary algorithms in detecting and eliminating the current harmonics.

## Supporting information

S1 File (DOCX)

S2 File (DOCX)
