# Supplementary material for: A developed DQ control method for shunt active power filter to improve power quality in transformers
Source: PLoS One. 2024 Jul 18;19(7):e0299635. doi: 10.1371/journal.pone.0299635 (PMC11257387; doi:10.1371/journal.pone.0299635)
Supplement: S2 File — (DOCX) [file pone.0299635.s002.docx]

A developed DQ control method for shunt active power filter to improve power quality in transformers

Saad F. Al-Gahtani^1¶^ , Elbarbary Z. M. S. ^1*, 2¶^, and Shaik Mohammad Irshad^1¶^

^1^Electrical Engineering Department, College of Engineering, King Khalid University, Abha, Saudi Arabia.

^2^Electrical Engineering, Faculty of Engineering, Kafrelsheikh University, KafrelSheikh, Egypt

*****Corresponding author

E-mail: [albrbry@kku.edu.sa](mailto:albrbry@kku.edu.sa) (EZMS)

${THD}_{X}= \frac{\sqrt{\sum_{h=2}^{\infty} {X_{h,rms}}^{2}}}{X_{1}}\times100\%$ $X_{h,rms}$ $X_{1}$ $P_{T =}P_{NL} + P_{LL}$ $P_{T}$ $P_{NL}$ $P_{LL}$ $opf$ $opf= \frac{1}{\sqrt{\begin{aligned} \\ \\ \\ \\ \\ \\ 1+{THD}^{2} \end{aligned}}}DPF$ $DPF$

**Harmonic Mitigation Techniques:**

The performance of the shunt APF is evaluated by measuring the source currents THDs and amplitudes.


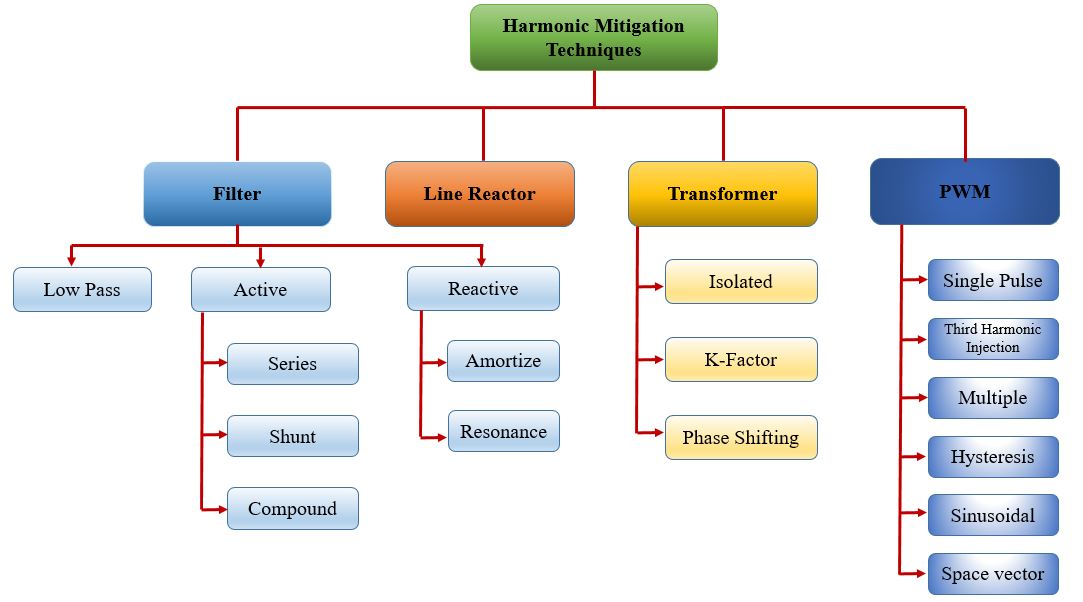


**Harmonic mitigation techniques.**

Table 1. **Features of several harmonic mitigation techniques.**

| **Type** | | **Advantages** | **Disadvantages** |
| --- | --- | --- | --- |
| **Filter** | **Passive/Low Pass** | - Cost-effective, low residual harmonics, - results can be predicted and guaranteed, - reduce cable heating and line loss(26,38) | Must be connected to load in series, can be used only with nonlinear loads, increases the heating effect, lower life of linear loads, leads to low power factor(26,38,39) |
|  | **Active** | Can mitigate up to 50^th^ order harmonics, can achieve less than 5% of distortion level(17,40) | Complex power electronic circuit, requires more maintenance, high cost and more loss compared to passive filters(17,40,41) |
| **Line Reactor** | **AC and DC** | Reduce surge currents, Simple and reduce good level of harmonics at reasonable price(17,26,39) | Develops voltage drop, increase system losses, Not effective in completely mitigating harmonic distortions(26) |
| **Transformer** | **isolated** | Effective in both common and normal mode disturbance with good circuit isolation (26) | Size of the isolated transformer should match the rated load current, increase circuit losses, and High cost compared to line reactor(26) |
|  | **K-Factor** | Ability to withstand the heat caused by eddy current losses, and the mixed (linear and nonlinear) load has a lower K-factor need than the nonlinear load(38). | Cost is high compared to an isolated transformer for each horsepower(26) |
|  | **Phase Shifting** | 5^th^ and 7^th^ order harmonics can be canceled at equal loading situation(17,38,41) | A bulky setup as two-phase shifting transformers are required, resulting in increase of losses(38,39) |
| **Pulse Width Modulation** | **Single Pulse** | Simple to implement, easy to use and control(26) | Presence of harmonic content due to the single pulse, slow dynamic performance(26) |
|  | **Multiple pulses** | Reliable and reduces THD less than 5% at loads.(3,26,38) | High installation costs. Though harmonics are reduced, there is a reduction in efficiency. One setup for each product. Large footprint is required.(26,38,41) |
|  | **Sinusoidal** | Simple computation for reference generation, Improves the distortion factor, and eliminates higher harmonics when used with filters.(26,41) | Modulation (chopping) frequency needs to be synchronized with the inverter output frequency, which has the effect of over-modulation.(38,42) |
|  | **Hysteresis** | - Ability to respond quickly to transients in load and line - easy to implement and robustness in load variation.(22,23) | Modulation frequency varies in a band for the fundamental frequency.(22,23) |
|  | **Space Vector** | Can achieve low THD, low computational losses by preventing unnecessary switching, better modulation. Optimized efficiency and highly reliable(20,43) | Reduction in volume and sometimes increase in switching losses(26) |
|  | **Third Harmonic Injection** | Advantage of better utilization of the available DC.  Inverter output voltage is raised by 15.5% without over modulation. Third harmonic can be completely eliminated.(26) | Higher order harmonics still exists(26) |

**Different types of synchronization techniques.**

**Synchronization techniques.**

To overcome these issues in traditional DQ, a modified DQ technique is proposed to control the shunt APF for minimizing the harmonics in the power transformers.

Proposed System

#### **System Configuration**

**Block Diagram of Proposed control scheme with Shunt APF control.**

Table 2. **System parameters.**

| **Details of Parameter** | | **Value** |
| --- | --- | --- |
| **AC Source voltage** | | 13.8kV |
| **Line Frequency** | | 60Hz |
| **Transformer** | **Rated Power** | 100kVA |
|  | **Connection** | Dyn11 |
|  | **Rated Frequency** | 50 or 60Hz |
|  | **HV** | 13.8kV |
|  | **LV** | 400V |
|  | **Voltage Impedance** | 4% |
|  | **No Load Losses** | 520W |
|  | **Load Losses** | 3200W |
| **Nonlinear Load** | 10kW | |
| **LC filter** | 2mH, C= 150μF, R_d_=0.2Ω | |
| **DC Voltage** | 900V | |

#### **Developed DQ control method**

$a$ $a^{2}$ $\alpha$ $\beta$ $a$ $a^{2}$ $\alpha=1\left( t-\frac{1}{90} \right), f=60 Hz$ $\beta=1\left( t-\frac{1}{180} \right), f=60 Hz$ $\left[ \begin{matrix} i^{(0)} \\ i^{(1)} \\ i^{(2)} \end{matrix} \right]=\frac{1}{3}\left[ \begin{matrix} 1 & 1 & 1 \\ 1 & \alpha& \beta\\ 1 & \beta& \alpha\end{matrix} \right]\left[ \begin{matrix} i_{La} \\ i_{Lb} \\ i_{Lc} \end{matrix} \right]$ ${i_{L}(t)}^{(1)}=\frac{1}{3} (i_{La}(t)+\alpha i_{Lb}(t)+\beta i_{Lc}(t))$ ${i_{La}(t)}^{(1)}={i_{L}(t)}^{(1)}$ ${i_{Lb}(t)}^{(1)}={\beta i_{L}(t)}^{(1)}$ ${i_{Lc}(t)}^{(1)}=\alpha{i_{L}(t)}^{(1)}$ $\left[ \begin{matrix} {i_{La}(t)}^{(2)} \\ {i_{Lb}(t)}^{(2)} \\ {i_{Lc}(t)}^{(2)} \end{matrix} \right]=\left[ \begin{matrix} i_{La}(t) \\ i_{Lb}(t) \\ i_{Lc}(t) \end{matrix} \right]-\left[ \begin{matrix} {i_{La}(t)}^{(1)} \\ {i_{Lb}(t)}^{(1)} \\ {i_{Lc}(t)}^{(1)} \end{matrix} \right]$ $\left[ \begin{matrix} i_{d}^{(2)} \\ i_{q}^{(2)} \end{matrix} \right]=\frac{2}{3}\left[ \begin{matrix} \cos\theta& \cos\left( \theta-120^{\circ} \right) & \cos\left( \theta+120^{\circ} \right) \\ -\sin\theta& -sin \left( \theta-120^{\circ} \right) & -sin \left( \theta+120^{\circ} \right) \end{matrix} \right]\left[ \begin{matrix} i_{La} \\ i_{Lb} \\ i_{Lc} \end{matrix} \right]$ $\theta=\omega t$ $\left[ \begin{matrix} i_{inja,ref} \\ i_{injb,ref} \\ i_{injc,ref} \end{matrix} \right]=\left[ \begin{matrix} \cos(\omega t+\theta) & -\sin(\omega t+\theta) \\ \cos\left( \omega t+\theta-\frac{2\pi}{3} \right) & -sin \left( \omega t+\theta-\frac{2\pi}{3} \right) \\ \cos\left( \omega t+\theta+\frac{2\pi}{3} \right) & -sin \left( \omega t+\theta+\frac{2\pi}{3} \right) \end{matrix} \right]\left[ \begin{matrix} i_{d}^{(2)} \\ i_{q}^{(2)} \end{matrix} \right]$The measured current from the shunt APF is compared to the reference current. A hysteresis current control is used to control and generate the firing signals for IGBT switches of the inverter switches as depicted in Figure below.

**(a)**

**(b)**

**Block diagram of DQ control methods (a) the traditional, and (b) the developed.**

**Hysteresis current controller.**

**Obtained Results:**

Magnitudes and THDs of transformer voltage and current.

| **Parameter** | | **Without Shunt APF** | **Conventional DQ** | **Developed DQ** |
| --- | --- | --- | --- | --- |
| **V_abc_TR_1_** | **V_peak_** | 11267.65, 11267.65, 11267.65 | 11267.65, 11267.65, 11267.65 | 11267.65, 11267.65, 11267.65 |
|  | **THD** | 0.01, 0.01, 0.01 | 0.01, 0.01, 0.01 | 0.01, 0.01, 0.01 |
| **V_abc_TR_2_** | **V_peak_** | 326.15, 326.15, 326.15 | 328.3, 328.3, 328.3 | 326.5, 326.5, 326.5 |
|  | **THD** | 3.2, 3.2, 3.2 | 1.5, 1.5, 1.5 | 1.5, 1.7, 1.6 |
| **I_abcTR_2_** | **A_peak_** | 1.08, 1.08, 1.08 | 0.98, 0.98, 0.98 | 1.13, 1.13, 1.13 |
|  | **THD** | 28, 28, 28 | 5.1, 5.15, 5.2 | 4.3, 4.4, 3.7 |
| **I_abc_TR_2_** | **A_peak_** | 37, 37, 37 | 33.85, 33.8, 33.86 | 38.98, 38.97, 38.95 |
|  | **THD** | 28.13, 28.13, 28.13 | 5.14, 5.1, 5.2 | 3.9, 4, 4 |

**Efficiency and overall power factor of the system.**

| **Parameters** | | **θ_a_ (°)** | **θ_b_ (°)** | **θ_c_ (°)** | **P_primary_ and P_secondary_ (kW)** | **η (%)** | **PF** |
| --- | --- | --- | --- | --- | --- | --- | --- |
| **Without Shunt APF** | **V_abc_TR_1_** | 0 | 240 | 120 | 18.17 | 99.2 | 0.96 |
|  | **I_abcTR_1_** | -5.6 | 234.4 | 114.4 |  |  |  |
|  | **V_abc_TR_2_** | -30.7 | 209.3 | 89.3 | 18.04 |  | 0.96 |
|  | **I_abc_TR_2_** | -35.4 | 204.6 | 84.6 |  |  |  |
| **Conventional DQ** | **V_abc_TR_1_** | 0 | 240 | 120 | 14.5 | 99.6 | 0.88 |
|  | **I_abcTR_1_** | 28.8 | 268.9 | 148.9 |  |  |  |
|  | **V_abc_TR_2_** | -30.8 | 209.2 | 89.2 | 14.45 |  | 0.87 |
|  | **I_abc_TR_2_** | -0.9 | 239.1 | 119.2 |  |  |  |
| **Developed DQ** | **V_abc_TR_1_** | 0 | 240 | 120 | 19.09 | 99.9 | 0.99 |
|  | **I_abcTR_1_** | -1.3 | 238.7 | 118.8 |  |  |  |
|  | **V_abc_TR_2_** | -30.9 | 209.1 | 89.1 | 19.08 |  | 0.99 |
|  | **I_abc_TR_2_** | -31.1 | 208.9 | 88.9 |  |  |  |
