# Supplementary material for: A developed DQ control method for shunt active power filter to improve power quality in transformers
Source: PLoS One. 2024 Jul 18;19(7):e0299635. doi: 10.1371/journal.pone.0299635 (PMC11257387; doi:10.1371/journal.pone.0299635)
Supplement: S1 File — (DOCX) [file pone.0299635.s001.docx]

To
Academic Editor,

PLOS ONE

**Subject: Regarding manuscript ID manuscript # PONE-D-23-32919R3**

**Article Title: "** **A developed DQ control method for shunt active power filter to improve power quality in transformers"**

**Dear Prof.,**

The authors would like to express their sincere gratitude to the Editor’s constructive comments and valuable suggestions to improve the article. The author has revised the paper according to the editorial directions. The responses to the editor comments can be found below their respective comments, and the changes can be tracked in the manuscript.

**Additional Editor Comments:**

Response to the Editor Comments: All the comments by the reviewer are addressed and the necessary changes are made in the manuscript.

**Response to Editor’s Comments**

**Comment_1:**

1. There is a difference between the author names listed in the submission and your manuscript file.An author is listed as [Elbarbary Z. M. S.] in the manuscript file but as [zakaria elbarbary] in the online submission form. If the name is incorrect in the manuscript, please correct this. If the name is incorrect in the submission form, please let us know so we can correct the author’s profile for you.

Please note that the use of middle initials/names should be consistent in the manuscript’s author list, the submission form, and the author contributions initials. Furthermore, author names in the byline in your manuscript should also include the author's first names before their surnames. The author names will be typeset exactly as they appear in the manuscript so please ensure that the information provided is correct.

**Response:**

**Authors appreciate editor for his valuable direction. It is to inform that the correct name is Z. M. S. Elbarbary. (as present in revised manuscript). We request the editorial team to make the changes in the online submission form as Z. M. S. Elbarbary.**

**Comment 2:**

2. Your citations and Reference list must be numbered and in the order that they appear in the text. In the text, cite the reference number in square brackets (e.g., “We used the techniques developed by our colleagues [19] to analyze the data”).

Please modify your References section so that it is in ascending numerical order, free from any duplicates or missing reference numbers. For further information, please see our guidelines for references: http://journals.plos.org/plosone/s/submission-guidelines#loc-references.

If you are using EndNote, please ensure that you use the most recent version of the PLOS output style, which can be found at

<http://endnote.com/downloads/style/plos-public-library-science-all-journals>"

**Response:**

**As per the suggestion the references are rechecked and arranged in order.**

**Comment 3:**

Funding information for your study should not appear in the Acknowledgments section or other areas of your text, but must be entered into the online Editorial Manager in the Financial Disclosure field. The Financial Disclosure in your published article will be derived from the information provided in the system.
Please remove any funding information from your manuscript, and ensure that the Financial Disclosure statement provided in the submission system is correct.

**Response:**

**Authors appreciate the editor’s instruction.**

**Funding information is removed from the manuscript.**

**Comment 4:**

We note that the Funding Statement given in your manuscript does not match the Funding Statement given in the submission system. Funding statements should not be included within the manuscript file, therefore, please remove this statement from the manuscript file and ensure that the statement is correct in the submission system.

**Response:**

**As per the suggestion, the funding information is removed from the manuscript and correctly presented in the submission system. The funding information should be presented as “The author extends his appreciation to the Deanship of Scientific Research at King Khalid University for supporting this work through General Research Project under grant number (RGP.1/425/44).”**

**Thank you very much for your cooperation.**

**Sincerely yours.**

*Authors*
