# Supplementary material for: A developed DQ control method for shunt active power filter to improve power quality in transformers
Source: PLoS One. 2024 Jul 18;19(7):e0299635. doi: 10.1371/journal.pone.0299635 (PMC11257387; doi:10.1371/journal.pone.0299635)
Supplement: Attachment — Submitted filename: Article_Transformer_yellow.docx [file pone.0299635.s003.docx]

A developed DQ control method for shunt active power filter to improve power quality in transformers

Saad F. Al-Gahtani^1¶^ , Elbarbary Z. M. S. ^2¶^, and Shaik Mohammad Irshad^1¶^

^1^Electrical Engineering Department, College of Engineering, King Khalid University, Abha, Saudi Arabia.

^2^Electrical Engineering, Faculty of Engineering, Kafrelsheikh University, Kafrel-Sheikh, Egypt

*****Corresponding author

E-mail: [zsalbarbary@](mailto:zsalbarbary@)gmail.com (EZMS)

Abstract

Power transformers are the most important component in power system. Exposing these transformers to the harmonic distortions causes additional heat losses, insulation stress, decrease in lifetime of insulation, and reduced power factor with decrease in efficiency of the system. The lifespan of distribution transformers is influenced by the fragility of power quality in power networks. Harmonic mitigation filters with robust control technique are required to reduce the harmonic effects on power transformer. Traditionally, synchronous reference frame (DQ) is employed to control the shunt active power filter (APF) for mitigation of harmonics in power transformers. DQ control of Shunt APF lacks merits of fast response, delayed operation due to phased lock loop under abnormal grid conditions leading to insufficient harmonic elimination. A developed DQ method based on detecting the positive and negative sequence components is proposed to precisely control the shunt APF for reliable operation of power transformer. This detection technique improves the response time, mitigate the harmonics effecting the operation of transformer and overall power factor. The proposed control system is evaluated under different abnormal operating scenarios and compared with traditional DQ method. The results and analysis confirm the efficacy of the developed DQ method in improving the power transformer performance.

Key Words: DQ control, Harmonic distortion, Power quality, Shunt active power filter, Transformer losses.

Introduction

Power quality refers to the characteristics of electrical power that affect the performance and reliability of electrical equipment and systems. It involves various parameters, including voltage, frequency, waveform shape, harmonics, transients, and interruptions. The purpose of power quality is to deliver electrical power to end-users within specified limits and without significant disturbances that can cause equipment malfunction or operational problems(1,2). Harmonic effect on the power system is one of the most frequently addressed issues for any power system. Some abnormal components are effective and dangerous, whereas others are insignificant(3). The characteristics of the power supply have been affected by interruptions on the supply or load side. Inadequate power quality causes sensitive equipment to malfunction, leading to monetary, energy, and productivity losses(1,2). In a modern distribution system, power quality monitoring has become essential.

The rise of composite electrical equipment and the emerging use of solid-state nonlinear loads are the primary drivers of escalating power quality issues(4–6). These elements lower system efficiency, lead to supply voltage distortion, and degrade the performance of other loads on the same distribution system.

Transformers are known as the core of a power system. Transformers are highly efficient and intended to handle linear loads at power frequency (50/60 Hz)(7,8). However, with the development of power electronic equipment, the number of non-linear loads has increased significantly. These devices frequently bring harmonic currents into the system and distorting the voltage waveform(9). As a result, these devices would have a direct impact on the distribution transformer, which is designed to serve linear loads at a rated frequency. Temperature increases are expected as a result of increased core and winding losses caused by voltage and current harmonics(3,7). If the temperature at the winding hottest-spot rises over a typical value, the insulation may degrade, resulting in a reduced lifetime and an earlier transformer breakdown(3,7,10). Several studies have demonstrated that harmonics can cause serious difficulties led to a variety of losses, including economic, human, and equipment life(2,11,12). Consequently, research into harmonic effects has risen in order to develop acceptable solutions that can eliminate or lessen harmonic effects on transformers(13).

Concern has therefore increased over the severe effects of harmonics. The maximum permitted harmonic injection from the customer to the point of common coupling (PCC) and the overall harmonic distortion applied to the power system have been determined in accordance with IEC Standard 61000-3-6(14) and IEEE Standard 519-2014(15). The capacity of distribution transformers is frequently derated to prolong the life of the transformers. The IEEE Standard C57.110-2008 has been created in order to de-rate distribution transformers having voltage ratings up to 69kV (15,16).

In this article, the influence of current harmonics on power transformers is investigated through mathematical model and analysis. Then, mitigation techniques are investigated to identify the merits and demerits of each control technique. Among these techniques, a shunt APF has the ability to eliminate the power quality issues related to the currents(17,18). To overcome the issues of shortage in performance of traditional DQ, a developed DQ method is implemented to control the shunt APF. A detection technique of positive and negative sequence components is integrated in the traditional DQ method. As a result of this integration, the developed DQ method avoids the use of the low-pass or high-pass filters which delay the control response. In addition, it enhances the performance of the phase-locked loop (PLL) in determining the phase angle of the grid voltage (θ) required for synchronization under abnormal grid conditions. This will allow adequate compensations of the currents from the shunt APF (19,20). The key features of are enhancing PLL functionality under abnormal grid conditions which allows fast and reliable synchronization with less computational effort of the control system, improving the elimination of current harmonics, providing nearly unity overall power factor, and maintaining almost the ideal transformation ratio of the transformer. Detailed Comparative performance analysis between the traditional and developed DQ methods is presented to demonstrate the superiority of the developed DQ control method.

The remaining paper is organized as follows: Literature review on harmonic effects on transformers, overall power factor’s relationship with displacement power factor, different harmonic mitigation techniques, and control of shunt APF are provided in Section II. Section III provides the configuration of proposed system, developed DQ control method. Section IV presents the results and discussion on the outcomes of the proposed system. Finally, Section V provides the conclusions of this paper.

Literature review

A. Harmonics Effects on Transformers

Identifying the sources of harmonics is required to evaluate their effects on transformers and other power system equipment(2,12,13). Common harmonic sources include back-back diodes used in the public streetlamps, AC/DC converters, induction heating technology with power electronic switches, and passive power static compensation.  In a practical and under rated operating conditions, a transformer normally introduces low distortion. However, if the voltage is deviated beyond the rating of the transformer, the ferro-magnetic core will be under saturation conditions leading to the high distortion in the voltage and current parameters of the circuit. Hence the transformer will slide into the non-linear mode of operation producing harmonics.

The difference between input and output power of a transformer is transformed into heat in core and winding. This heat is dissipated from the transformer's exposed surfaces by a combination of convection and radiation(12,21,22). As a result, both the core's total surface area exposed and the windings affect how much heat is dissipated. Accurate forecasting of transformer temperature growth is difficult. One approach is to add the losses from the core and the winding assembly together and assume that the thermal energy is dissipated uniformly across the surface area of the assembly at all ambient temperatures. This is a reasonable assumption that the bulk of the transformer's surface area is the ferrite core area rather than the winding area.

To evaluate the harmonics effect on a transformer, the total harmonic distortion and transformer derating factor are essential (11).The total harmonic distortion (THD) indicates the amount of distortions in the voltage or the current and it can be represented as:

${THD}_{X}= \frac{\sqrt{\sum_{h=2}^{\infty} {X_{h,rms}}^{2}}}{X_{1}}\times100\%$	(1)

where $X_{h,rms}$ is the rms value of voltage or current harmonics, and $X_{1}$ is the fundamental voltage or current. Both voltage and current harmonics affect transformer during no-load and load conditions(3,11). Eddy and hysteresis currents losses are the cause of no-load effect (core losses). Ohmic (copper losses), eddy current, and stray losses are the loading effect which leads to the increase of oil temperature(23).  Additional consequences could include reduced nominal power and transformer lifetime(24).  The transformer's overall losses can be stated as follows (12,13,15,25):

$P_{T =}P_{NL} + P_{LL}$	(2)

where $P_{T}$ is the total losses, $P_{NL}$ is the no-load losses and $P_{LL}$ is the load losses of the transformer. The losses that are caused by harmonics either on the core or on the winding result in increasing the heat in the winding because of increasing the values of the voltages and the currents much higher than the rated values(26,27). Consequently, the heat starts damaging the insulation of the transformers. If the heat continues, there will be no more insulation in the transformer. Thus, the transformer is no more functioning. Thermal stresses which is generated by nonlinear load are responsible for about 50% of transformer’s loss of life. In addition, the harmonics impact the power factor of the power system(28,29). As harmonics increase in the system, the overall power factor ($opf$) decreases as seen in the below equation(2):

$opf= \frac{1}{\sqrt{1+{THD}^{2}}}DPF$	(3)

where $DPF$ is the displacement power factor. The effect of DPF and THD on opf are depicted in Fig 1. All of these harmonic effects, in addition to transformer life, will have an impact on power transformer efficiency. Therefore, it is critical to reduce the amount of harmonics in transformers to perform efficiently and last longer.

**(a)**

**(b)**

Fig 1. Effect on overall power factor Against (a) DPF and (b) THD.

B. Mitigation Techniques of Harmonic Effects on Transformer

Numerous mitigation schemes for power systems are put forward to reduce the adverse impacts of harmonics(11,30,31). Different types of mitigation techniques are listed in Fig 2. The merits and demerits of these mitigation techniques are presented in Table 1.

The development of APF allows simultaneous harmonic and reactive power compensation(32). Regardless of whether the load is non-linear or in an unbalanced state, the APF has the capacity to maintain the primary current balance after mitigating(17,33). Due to the fact that most industrial applications need current harmonics correction, shunt active filters are more prevalent than series filters(18,34). By providing a compensation current, shunt APF seeks to reduce level of harmonics and reactive power components of the source current(18,25,35–37). The performance of the shunt APF is evaluated by measuring the source currents THDs and amplitudes.

Fig 2. **Harmonic mitigation techniques.**

Table 1. **Features of several harmonic mitigation techniques.**

| **Type** | | **Advantages** | **Disadvantages** |
| --- | --- | --- | --- |
| **Filter** | **Passive/Low Pass** | - Cost-effective, low residual harmonics, - results can be predicted and guaranteed, - reduce cable heating and line loss(25,37) | Must be connected to load in series, can be used only with nonlinear loads, increases the heating effect, lower life of linear loads, leads to low power factor(25,37,38) |
|  | **Active** | Can mitigate up to 50^th^ order harmonics, can achieve less than 5% of distortion level(17,39) | Complex power electronic circuit, requires more maintenance, high cost and more loss compared to passive filters(17,39,40) |
| **Line Reactor** | **AC and DC** | Reduce surge currents, Simple and reduce good level of harmonics at reasonable price(17,25,38) | Develops voltage drop, Increase system losses, Not effective in completely mitigating harmonic distortions(25) |
| **Transformer** | **Isolated** | Effective in both common and normal mode disturbance with good circuit isolation (25) | Size of the isolated transformer should match the rated load current, Increase circuit losses, and High cost compared to line reactor(25) |
|  | **K-Factor** | Ability to withstand the heat caused by eddy current losses, and the mixed (linear and nonlinear) load has a lower K-factor need than the nonlinear load(37). | Cost is high compared to an isolated transformer for each horsepower(25) |
|  | **Phase Shifting** | 5^th^ and 7^th^ order harmonics can be canceled at equal loading situation(17,37,40) | A bulky setup as two phase shifting transformers are required, resulting in increase of losses(37,38) |
| **Pulse Width Modulation** | **Single Pulse** | Simple to implement, easy to use and control(25) | Presence of harmonic content due to the single pulse, slow dynamic performance(25) |
|  | **Multiple pulses** | Reliable and reduces THD less than 5% at loads.(3,25,37) | High installation costs. Though harmonics are reduced, there is a reduction in efficiency. One setup for each product. Large footprint is required.(25,37,40) |
|  | **Sinusoidal** | Simple computation for reference generation, Improves the distortion factor, and eliminates higher harmonics when used with filters.(25,40) | Modulation (chopping) frequency needs to be synchronized with the inverter output frequency, which has the effect of over-modulation.(37,41) |
|  | **Hysteresis** | - Ability to respond quickly to transients in load and line - easy to implement and robustness in load variation.(21,22) | Modulation frequency varies in a band for the fundamental frequency.(21,22) |
|  | **Space Vector** | Can achieve low THD, low computational losses by preventing unnecessary switching, better modulation. Optimized efficiency and highly reliable(19,42) | Reduction in volume and sometimes increase in switching losses(25) |
|  | **Third Harmonic Injection** | Advantage of better utilization of the available DC.  Inverter output voltage is raised by 15.5% without over modulation. Third harmonic can be completely eliminated.(25) | Higher order harmonics still exists(25) |

C. Control of Shunt APF

Control of shunt APF is influenced by the approaches used to generate command signals and controller, which controls switching pulse generation(17). Several studies presented different control strategies. The effective two time-domain control methodologies are instantaneous reactive power (IRP) and synchronous reference (DQ) coordinate system approaches(38,39,41). The extended IRP, the optimal-linear-prediction theorem, and the division frequency theorem are all dependent on modifying the IRP(43). To extract the average or oscillating components of the real (P) and reactive (Q) power, some control systems use a low-pass filter (LPF) or a high-pass filter (HPF)(17,38,41). Although LPFs and HPFs are simple to implement, but they induce phase delays at the fundamental frequency, which may compromise the shunt APF's performance. For extracting fundamental components, the LPFs are replaced with a PLL. However, PLL performs poorly under the conditions of unbalanced or distorted grid voltages(17,41). Fig 3 presents different types of synchronization techniques(17,25,37).

Synchronization Techniques

Single-phase

ZCD

PLL

ANN/ADALINE

Three-phase

ZCD

ANN/ADALINE

PLL

Unit Vector

Fundamental Component Extraction

Fig 3. **Synchronization techniques.**

To overcome these issues in traditional DQ, a modified DQ technique is proposed to control the shunt APF for minimizing the harmonics in the power transformers.

Proposed System

#### a) **System Configuration**

A power system is a complex circuit consisting of fundamental components including three-phase(3Φ) source connected to the nonlinear load (a resistive load supplied from 3Φ diode rectifier), through a 3Φ transformer of DYn11 vector group used commonly in distribution network. The nonlinear load reflects harmonic into the transformer increasing the losses. To mitigate the effect on loss level, a shunt APF circuit is inserted(44). The shunt APF consists of a three-phase inverter, a DC power supply, and a three-phase LC switching-harmonic filter. The considered electrical system network diagram and parameters are shown in Fig 4 and Table 2.

Fig 4. **Block Diagram of Proposed control scheme with Shunt APF control.**

Table 2. **System parameters.**

| **Details of Parameter** | | **Value** |
| --- | --- | --- |
| **AC Source voltage** | | 13.8kV |
| **Line Frequency** | | 60Hz |
| **Transformer** | **Rated Power** | 100kVA |
|  | **Connection** | Dyn11 |
|  | **Rated Frequency** | 50 or 60Hz |
|  | **HV** | 13.8kV |
|  | **LV** | 400V |
|  | **Voltage Impedance** | 4% |
|  | **No Load Losses** | 520W |
|  | **Load Losses** | 3200W |
| **Nonlinear Load** | 10kW | |
| **LC filter** | 2mH, C= 150μF, R_d_=0.2Ω | |
| **DC Voltage** | 900V | |

#### b) **Developed DQ control method**

The proposed control method of the shunt APF is based on modified DQ method as presented in Fig 5. This method depends on appearing of the negative sequence components in load current during abnormal conditions. In the traditional DQ method, the PLL has a poor performance under severe harmonic distortion. Additionally, LPFs add delay to the injected signals. For that, the proposed control method combines the traditional DQ method and a detection technique of negative sequence components to enhance the performance of the PLL as well as to increase the elimination of harmonics.

##### (1) ***Detection Technique***

The detection of the negative sequence components is based on the transformation of symmetrical components(19). This technique is performed in the time domain by converting the phasor rotation operators $a$ and $a^{2}$ to function of time. $\alpha$ and $\beta$ are time-domain operator representing $a$ and $a^{2}$.

$\alpha=1\left( t-\frac{1}{90} \right), f=60 Hz$	(4)

$\beta=1\left( t-\frac{1}{180} \right), f=60 Hz$	(5)

The positive sequence components of load currents are calculated; then, negative sequence components are mathematically detected by subtracting the three-phase quantities from the positive sequence components. The transformation of the symmetrical components is expressed as follows:

$\left[ \begin{matrix} i^{(0)} \\ i^{(1)} \\ i^{(2)} \end{matrix} \right]=\frac{1}{3}\left[ \begin{matrix} 1 & 1 & 1 \\ 1 & \alpha& \beta\\ 1 & \beta& \alpha\end{matrix} \right]\left[ \begin{matrix} i_{\mathrm{La}} \\ i_{\mathrm{Lb}} \\ i_{\mathrm{Lc}} \end{matrix} \right]$	(6)

The positive sequence of the load currents is as follows:

${i_{L}(t)}^{(1)}=\frac{1}{3} (i_{\mathrm{La}}(t)+\alpha i_{\mathrm{Lb}}(t)+\beta i_{\mathrm{Lc}}(t))$	(7)

Then, the positive sequence components of the load currents are as follows:

${i_{\mathrm{La}}(t)}^{(1)}={i_{L}(t)}^{(1)}$, ${i_{\mathrm{Lb}}(t)}^{(1)}={\beta i_{L}(t)}^{(1)}$, ${i_{\mathrm{Lc}}(t)}^{(1)}=\alpha{i_{L}(t)}^{(1)}$	(8)

Now, the negative sequence components can be found as:

$\left[ \begin{matrix} {i_{\mathrm{La}}(t)}^{(2)} \\ {i_{\mathrm{Lb}}(t)}^{(2)} \\ {i_{\mathrm{Lc}}(t)}^{(2)} \end{matrix} \right]=\left[ \begin{matrix} i_{\mathrm{La}}(t) \\ i_{\mathrm{Lb}}(t) \\ i_{\mathrm{Lc}}(t) \end{matrix} \right]-\left[ \begin{matrix} {i_{\mathrm{La}}(t)}^{(1)} \\ {i_{\mathrm{Lb}}(t)}^{(1)} \\ {i_{\mathrm{Lc}}(t)}^{(1)} \end{matrix} \right]$	(9)

##### (2) ***Control Method***

The detection method is applied on the measured load currents. A PLL is used with the measured voltages on point of common coupling. The negative components of the load currents are converted into synchronous dq coordinates by implementing Park’s transformation:

$\left[ \begin{matrix} i_{d}^{(2)} \\ i_{q}^{(2)} \end{matrix} \right]=\frac{2}{3}\left[ \begin{matrix} \cos\theta& \cos\left( \theta-120^{\circ} \right) & \cos\left( \theta+120^{\circ} \right) \\ -\sin\theta& -sin \left( \theta-120^{\circ} \right) & -sin \left( \theta+120^{\circ} \right) \end{matrix} \right]\left[ \begin{matrix} i_{\mathrm{La}} \\ i_{\mathrm{Lb}} \\ i_{\mathrm{Lc}} \end{matrix} \right]$	(10)

where $\theta=\omega t$ is angular position determined by the PLL. The inverse Park's transformation is then used to transform the negative sequence components of the shunt APF into the abc coordinate’s frame of reference:

$\left[ \begin{matrix} i_{inja,ref} \\ i_{injb,ref} \\ i_{injc,ref} \end{matrix} \right]\boldsymbol{=}\left[ \begin{matrix} \cos(\omega t+\theta) & -\sin(\omega t+\theta) \\ \cos\left( \omega t+\theta-\frac{2\pi}{3} \right) & -sin \left( \omega t+\theta-\frac{2\pi}{3} \right) \\ \cos\left( \omega t+\theta+\frac{2\pi}{3} \right) & -sin \left( \omega t+\theta+\frac{2\pi}{3} \right) \end{matrix} \right]\left[ \begin{matrix} i_{d}^{(2)} \\ i_{q}^{(2)} \end{matrix} \right]$	(11)

The measured current from the shunt APF is compared to the reference current. A hysteresis current control is used to control and generate the firing signals for IGBT switches of the inverter switches as depicted in Fig 6.

**(a)**

**(b)**

Fig 5. **Block diagram of DQ control methods (a) the traditional, and (b) the developed.**

Fig 6. **Hysteresis current controller.**

Results and discussion

The system shown in Figs 4-6 with aid of equations (1-15) is simulated to evaluate the control system response with and without the proposed APF using Matlab/Simulink. Figs 7-12 show waveforms of the system variables with and without shunt APF. In addition, analysis of the system are provided in Tables 3, 4 and 5. The analysis are obtained using Simulink steady state and FFT Analysis.

As displayed in Figs 7-10, the voltages and currents at the primary and secondary sides of the transformer with and without shunt APF. The currents are balanced and sinusoidal with the shunt APF. However, the voltage and current with the proposed control method have significant improvement compared to that of traditional method in terms of the THD and phase displacement between the voltage and current as shown in Table 3. Without the shunt APF, the current THD is about 28% at both sides of the transformer. This amount of THD has considerably decreased to about 5% under traditional control method and about 4% under the developed control method meeting the acceptable levels as per IEEE standards. Moreover, a proper synchronization between the phase voltage and phase current at 1 and 2 windings of the transformer is achieved using the modified DQ control method. The waveforms and values of the corresponding voltage and currents are presented in Fig 11 and Table 4; The synchronization and THD values from Table 3 and 4 has shown the effectiveness of the developed control method in improving the overall power factor. As a result, the use of modified control method has increased the capacity transferred active power and has decreased the wasted or lost active power as depicted in Fig 12 and Table 4. The power efficiency of the transformer under the developed control method is also the highest among other systems.

Fig 7. **Primary and secondary voltages and currents with nonlinear load of 10kW and without the shunt APF.**

Fig 8. **Transformer primary and secondary voltages with the shunt APF controlled by traditional DQ method (on the left) and by developed DQ method (on the right).**

Fig 9. **Transformer primary and secondary currents with the shunt APF controlled by traditional DQ method (on the left) and by developed DQ method (on the right).**

Fig 10. **Load currents and compensated currents from the shunt APF controlled by traditional DQ method (on the left) and by developed DQ method (on the right).**

**(a)**

**(b)**

Fig 11. **Tracking the phase angle of (a) primary voltage and (b) secondary voltage.**

**(a)**

**(b)**

Fig 12. **Power of (a) primary side and (b) secondary side.**

Table 3. Magnitudes and THDs of transformer voltage and current.

| **Parameter** | | **Without Shunt APF** | **Conventional DQ** | **Developed DQ** |
| --- | --- | --- | --- | --- |
| **V_abc_TR_1_** | **V_peak_** | 11267.65, 11267.65, 11267.65 | 11267.65, 11267.65, 11267.65 | 11267.65, 11267.65, 11267.65 |
|  | **THD** | 0.01, 0.01, 0.01 | 0.01, 0.01, 0.01 | 0.01, 0.01, 0.01 |
| **V_abc_TR_2_** | **V_peak_** | 326.15, 326.15, 326.15 | 328.3, 328.3, 328.3 | 326.5, 326.5, 326.5 |
|  | **THD** | 3.2, 3.2, 3.2 | 1.5, 1.5, 1.5 | 1.5, 1.7, 1.6 |
| **I_abcTR_2_** | **A_peak_** | 1.08, 1.08, 1.08 | 0.98, 0.98, 0.98 | 1.13, 1.13, 1.13 |
|  | **THD** | 28, 28, 28 | 5.1, 5.15, 5.2 | 4.3, 4.4, 3.7 |
| **I_abc_TR_2_** | **A_peak_** | 37, 37, 37 | 33.85, 33.8, 33.86 | 38.98, 38.97, 38.95 |
|  | **THD** | 28.13, 28.13, 28.13 | 5.14, 5.1, 5.2 | 3.9, 4, 4 |

Table 4. **Efficiency and overall power factor of the system.**

| **Parameters** | | **θ_a_ (°)** | **θ_b_ (°)** | **θ_c_ (°)** | **P_primary_ and P_secondary_ (kW)** | **η (%)** | **PF** |
| --- | --- | --- | --- | --- | --- | --- | --- |
| **Without Shunt APF** | **V_abc_TR_1_** | 0 | 240 | 120 | 18.17 | 99.2 | 0.96 |
|  | **I_abcTR_1_** | -5.6 | 234.4 | 114.4 |  |  |  |
|  | **V_abc_TR_2_** | -30.7 | 209.3 | 89.3 | 18.04 |  | 0.96 |
|  | **I_abc_TR_2_** | -35.4 | 204.6 | 84.6 |  |  |  |
| **Conventional DQ** | **V_abc_TR_1_** | 0 | 240 | 120 | 14.5 | 99.6 | 0.88 |
|  | **I_abcTR_1_** | 28.8 | 268.9 | 148.9 |  |  |  |
|  | **V_abc_TR_2_** | -30.8 | 209.2 | 89.2 | 14.45 |  | 0.87 |
|  | **I_abc_TR_2_** | -0.9 | 239.1 | 119.2 |  |  |  |
| **Developed DQ** | **V_abc_TR_1_** | 0 | 240 | 120 | 19.09 | 99.9 | 0.99 |
|  | **I_abcTR_1_** | -1.3 | 238.7 | 118.8 |  |  |  |
|  | **V_abc_TR_2_** | -30.9 | 209.1 | 89.1 | 19.08 |  | 0.99 |
|  | **I_abc_TR_2_** | -31.1 | 208.9 | 88.9 |  |  |  |

Additionally, transformation ratio of the transformer is evaluated as shown in Table 5 where ideal transformation ratio is 34.5:1. Without the shunt APF, the ratio is 34.55:1 which indicates that the secondary voltage is reduced and secondary current is increased. This increase in secondary current leads to an increase in losses in winding leading to the rise in temperature of the secondary winding. This temperature increase effect the life of the transformer. With the shunt APF controlled by the traditional method, the ratio is 34.3 meaning secondary voltage is increased effecting the secondary winding insulation. Finally, the ratio with the developed DQ control is 34.51 which means the transformer is employed under ideal rated state of the transformer. Due to this the transformer will have the capability to withstand the loss and temperature effect.

Table 5. **Transformer ratio**

| **Condition of Transformer** | **Ratio** |
| --- | --- |
| Ideal Transformer | 34.50 |
| Without shunt APF | 34.55 |
| Shunt APF controlled by traditional DQ method | 34.30 |
| Shunt APF controlled by developed DQ method | 34.51 |

Conclusion

Harmonics are undesirable components that could affect power transformers' performance and lives. Two types of effects on transformers from the harmonics are no-load and load losses. No-load and load losses increase the heat of the transformer which consequently weakens the insulation of the transformer and reduces its lifespan. Therefore, eliminating the current harmonics and power quality issues before reaching the transformers is must.

Shunt APFs can provide a great solution for eliminating power quality issues associated with system currents. The study has introduced a developed DQ control method for shunt APF. The method contains an efficient technique in detecting the positive and negative sequence components. The technique improves the response and reliability of PLL synchronization and the capability of the shunt APF in removing the current harmonics. Moreover, this technique eliminates the use of LPFs which used in the traditional DQ method.

A comparative study between the traditional DQ control and the developed control were provided. The results has validated the superiorities of the developed DQ approach in minimizing reactive and harmonic components, synchronizing the phase angles between the voltage and currents, providing fast response, improving the transformer's transformation ratio and efficiency, better capacity of transferred active power, and maintaining almost unity power factor. For that, the developed DQ control method for shunt APF provides a protection wall for power transformers from power quality risks that threaten their performances or lives.

**Acknowledgement**

The author extends his appreciation to the Deanship of Scientific Research at King Khalid University for supporting funding this work through General Research Project under grant number (RGP.1/425/44).

**Competing interests**

The authors have declared that no competing interests exist.

# **References**

1.	Jaiswal GC, Ballal MS, Tutakne DR, Suryawanshi HM. Impact of Power Quality on the Performance of Distribution Transformers: A Fuzzy Logic Approach to Assessing Power Quality. IEEE Industry Applications Magazine. 2019 Sep 1;25(5):8–17.

2.	Hasan S, Daut I, Syafruddin HS, Ali R, Samila M, Haziah H. The Effects of Harmonic Components on Transformer Losses of Sinusoidal Source Supplying Non-Linear Loads. Am J Appl Sci. 2006;3(12):2131–3.

3.	Henderson RD, Rose PJ. Harmonics: The Effects on Power Quality and Transformers. Vol. 30, IEEE Transactions On Industry Applications. 1994.

4.	Suraya S, Sujatha P. A Novel Simplified Unit Vector Theory for Improvement of Voltage Quality in Distribution System. Science Direct. 2016;00:1–9.

5.	Jinia Roy, Akshay K Jain, Barry Mather. Impacts of Experimentally Obtained Harmonic Spectrums of Residential Appliances on Distribution Feeder. In: 2020 IEEE Texas Power and Energy Conference (TPEC) : date: February 06-February 07, 2020 : location: Memorial Student Center at Texas A & M University, 275 Joe Routt Blvd #2240, College Station, TX, USA. 2020.

6.	Yu J, Xu Y, Li Y, Liu Q. An Inductive Hybrid UPQC for Power Quality Management in Premium-Power-Supply-Required Applications. IEEE Access. 2020;8:113342–54.

7.	Awadallah MA, Xu T, Venkatesh B, Singh BN. On the Effects of Solar Panels on Distribution Transformers. IEEE Transactions on Power Delivery. 2016 Jun 1;31(3):1176–85.

8.	Suraya S, Sujatha P, Bharat Kumar P. Distributed generation with high voltage gain DC-DC converter integrated DVR as uninterrupted power supply. ARPN Journal of Engineering and Applied Sciences. 2018;13(9):3257–65.

9.	Arthur G, Carlos DA, Brand C, Agra R. Cascaded Open-End Winding Transformer Based DVR. 2018;54(2):1490–501.

10.	T. Dao, B.T. Phung, T.Blackburn. dao2015. In: 2015 IEEE PES Asia-Pacific Power and Energy Engineering Conference. 2016.

11.	M. Shareghi, B.T. Phung, M.S. Naderi, T.R. Blackburn, E. Ambikairajah. Effects of Current and Voltage Harmonics on Distribution Transformer Losses. In: International Conference on Condition Monitoring and Diagnosis (CMD), 2012 23-27 Sept 2012, [Sanur], Bali, Indonesia. 2012. p. 633–6.

12.	Said DM, Said DM, Nor KM. Effects of harmonics on distribution transformers Effects of Harmonics on Distribution Transformers’ [Internet]. 2009. Available from: https://www.researchgate.net/publication/224400789

13.	Mousavi S, Tavan B, Bakhtiari H. The Review and Analysis The destructive effects of harmonics on a Sample MV/LV Transformer. In: 20th International Conference on Electricity Distribution, CIRED . 2009. p. 8–11.

14.	M. McGranaghan, G. Beaulieu. Update on IEC 61000-3-6: Harmonic Emission Limits for Customers Connected to MV, HV, and EHV. In: IEEE. 2006. p. 1–4.

15.	IEEE Power and Energy Society. IEEE Recommended Practice and Requirements for Harmonic Control in Electric Power Systems Sponsored by the Transmission and Distribution Committee IEEE Power and Energy Society. In: IEEE Std 519 -2014. 2014.

16.	IEEE Standards Association. 1159-2019 - IEEE Recommended Practice for Monitoring Electric Power Quality. IEEE; 2019.

17.	Hoon Y, Radzi MAM, Zainuri MAAM, Zawawi MAM. Shunt active power filter: A review on phase synchronization control techniques. Vol. 8, Electronics (Switzerland). MDPI AG; 2019.

18.	Khan A, Jaffery MH, Javed Y, Arshad J, Rehman AU, Khan R, et al. Hardware-in-the-Loop Implementation and Performance Evaluation of Three-Phase Hybrid Shunt Active Power Filter for Power Quality Improvement. Math Probl Eng. 2021;2021.

19.	Al-Gahtani SF, Nelms RM. Performance of a shunt active power filter for unbalanced conditions using only current measurements†. Energies (Basel). 2021 Jan 2;14(2).

20.	Al-Gahtani SF, Abdulwase Bakr B, Azazi HZ, Irshad SM, Bhutto JK, Majahar HM, et al. A New Technique Implemented in Synchronous Stationary Frame (DQ) for DVR Control under Severe Sag and Swell Conditions. IEEE Access. 2022;

21.	Fatiha M, Mohamed M, Nadia AA. New hysteresis control band of an unified power quality conditioner. Vol. 81, Electric Power Systems Research. 2011. p. 1743–53.

22.	Arpitha M J, Sowmyashree N, M S Shashikala. Power Quality Enhancement using Dynamic Voltage Restorer ( DVR ) by Artificial Neural Network and Hysteresis Voltage Control Techniques. IEEE, Global Conference for Advancement in Technology (GCAT), Bangalore, India. 2019 Oct 18;3–8.

23.	T.L.G. Soh, D.M. Said, N. Ahmad, K.M. Nor, F. Salim. Experimental Study on the Impact of Harmonics on Transformer. In: 2013 IEEE 7th International Power Engineering and Optimization Conference (PEOCO2013), Langkawi, Malaysia 3-4 June 2013. 2013. p. 686–90.

24.	Elmoudi A, Lehtonen M, Nordman H. Effect of Harmonics on Transformers Loss of life. In: Conference Record of the 2006 IEEE International Symposium on Electrical Insulation. 2006. p. 408–11.

25.	Said DM, Nor KM. Simulation of the impact of harmonics on distribution transformers. In: PECon 2008 - 2008 IEEE 2nd International Power and Energy Conference. 2008. p. 335–40.

26.	El Sayed NG, El-Saady G, Ibrahim ENA, Mohamed MA. Dynamic Voltage Restorer for Enhancing Distribution Systems Power Quality. In: Proceedings of the International Japan-Africa Conference on Electronics, Communications and Computations, JAC-ECC 2019. Institute of Electrical and Electronics Engineers Inc.; 2019. p. 210–5.

27.	Preethy Thankachen, Daru Anna Thomas. Hysteresis Controller Based Fault Current Interruption Using DVR. International Conference on Magnetics, Machines & Drives (AICERA-2014 iCMMD). 2014.

28.	Said DM, Nor KM, Majid MS. Analysis of distribution transformer losses and life expectancy using measured harmonic data. In Institute of Electrical and Electronics Engineers (IEEE); 2010. p. 1–6.

29.	Mekhamer SF, Mohsen Ismael S, Abdelaziz Sherif M Ismael AY. Technical Comparison of Harmonic Mitigation Techniques for Industrial Electrical Power Systems [Internet]. 2012. Available from: https://www.researchgate.net/publication/280841080

30.	Harold Francisco Mazo Mantilla, Andres Pavas, Ivan Camilo Duran. Aging of Distribution Transformers due to Voltage Harmonics. In: 2017 IEEE Workshop on Power Electronics and Power Quality Applications (PEPQA) : proceedings : May 31 - June 2 , 2017, Bogotá, Colombia. 2017.

31.	Maheri HM, Vinnikov D, Chub A, Liivik E, Sidorov V. Impact of transformer turns ratio on the power losses and efficiency of the wide range isolated buck–boost converter for photovoltaic applications. Energies (Basel). 2020 Nov 1;13(21).

32.	Grabowski D, Maciążek M, Pasko M, Piwowar A. Time-invariant and time-varying filters versus neural approach applied to DC component estimation in control algorithms of active power filters. Appl Math Comput. 2018 Feb 15;319:203–17.

33.	Salih Mohammed Salih, Kaleid Waleed Abid, Munther Nayef. Practical Analysis of Harmonics Effects on Transformer. Elixir International Journal - Renewable Energy Engineering [Internet]. 2014 May 23;70:24361–7. Available from: https://www.researchgate.net/publication/262734983

34.	Shahzad M, Keerio MU, Shahzad Bajwa M. Modeling Of Factors Influencing Harmonic Distortion Based on Simulation Model of Personal Computer Factors influencing harmonic distortion View project Modeling Of Factors Influencing Harmonic Distortion Based on Simulation Model of Personal Computer. University of Sindh Journal of Information and Communication Technology (USJICT) [Internet]. 2020 Oct;4(3):173–80. Available from: https://www.researchgate.net/publication/348369526

35.	Gajanan C. Jaiswal, Makarand Sudhakar Ballal, Prasad A. Venikar, Hiralal M. Suryawanshi, D.R. Tutakne. Smart Transformers for Industrial Applications. In: 2017 IEEE Transportation Electrification Conference (ITEC-India). IEEE; 2017.

36.	Antonio Carlos Delaiba, Jose Carlos De Oliveira, Vilaca ALA, Cardoso JR. THE EFFECT OF HARMONICS ON POWER TRANSFORMERS LOSS OF LIFE. In: 38th Midwest Symposium on Circuits and Systems Proceedings [Internet]. 1995 [cited 2023 Jul 7]. p. 933–6. Available from: https://ieeexplore.ieee.org/document/510243

37.	Jyotsana Kaiwart, Uma P. Bala Raju. A study on harmonic mitigation by using isolation transformer. Research Journal of Engineering Sciences. 2017;6(9):29–31.

38.	Kazem HA. Harmonic mitigation techniques applied to power distribution networks. Vol. 2013, Advances in Power Electronics. 2013.

39.	Tugce Demirdelen, Mustafa Inci, K. Çagatay Bayindir, Mehmet Tumay. Review of Hybrid Active Power Filter        Topologies and Controllers. In: 4th International Conference on Power Engineering, Energy and Electrical Drives. IEEE; 2013. p. 587–92.

40.	Mortezaei A, Lute C, Simões MG, Marafão FP, Boglia A. PQ, DQ and CPT control methods for shunt active compensators - A comparative study. 2014 IEEE Energy Conversion Congress and Exposition, ECCE 2014. 2014;2994–3001.

41.	Sumant Kumar Dalai, Rojalin Sahu, Chandra Sekhar Tripathy. Harmonic Mitigation in Single -Phase Grid Connected Photovoltaic System Using SPWM Inverter. In: International Conference on Computational Intelligence for Smart Power System and Sustainable Energy (CISPSSE-2020) : (29-31, July 2020). 2020.

42.	Pakharia A, Gupta M. Dynamic Voltage Restorer for C Ompensation of Voltage Sag and Swell : a Literature Review. Int J Adv Eng Technol. 2012;4(1):347–55.

43.	Naif Thiyab M, Abbas Hasan M, waleed abid K. Effect of Harmonics on Power Transformers: A practical demonstration and analysis Dust Effect on PV Panel View project Biosensor View project Effect of Harmonics on Power Transformers: A practical demonstration and analysis [Internet]. Vol. 10, Article in Journal of Advanced Research in Dynamical and Control Systems. Special Issue; 2018. Available from: https://www.researchgate.net/publication/331473596

44.	Biricik S, Redif S, özerdem özgür C, Khadem SK, Basu M. Real-time control of shunt active power filter under distorted grid voltage and unbalanced load condition using self-tuning filter. IET Power Electronics. 2014 Jul 1;7(7):1895–905.
