# Supplementary material for: A developed DQ control method for shunt active power filter to improve power quality in transformers
Source: PLoS One. 2024 Jul 18;19(7):e0299635. doi: 10.1371/journal.pone.0299635 (PMC11257387; doi:10.1371/journal.pone.0299635)
Supplement: Attachment — Submitted filename: Reviewers comments.docx [file pone.0299635.s004.docx]

**Reviewer’s comments.**

1. There is a major drawback in the control methodology as the authors have ignored the zero-sequence components of the current which are essential parts when there are harmonics involved. No discussion is made in this regard.
2. No significant difference is observed in fig. 9 between the traditional method and the developed method.
3. Issues in the equation numbers as there are only 11 equations while the authors have said equations (1-15)
4. If the control methodology is strictly observed, there is a major flaw,the authors can be asked to justify why they ignored the zero-sequence components, if they have any justification.
5. Some of the issues are highlighted in yellow in the original doc file.
