# Supplementary material for: A developed DQ control method for shunt active power filter to improve power quality in transformers
Source: PLoS One. 2024 Jul 18;19(7):e0299635. doi: 10.1371/journal.pone.0299635 (PMC11257387; doi:10.1371/journal.pone.0299635)
Supplement: Attachment — Submitted filename: Response to Reviewers.docx [file pone.0299635.s005.docx]

**Professor Lalit Chandra Saikia, PhD**
Academic Editor,

PLOS ONE

**Subject: Regarding manuscript ID manuscript # PONE-D-23-32919**

**Article Title: "** **A developed DQ control method for shunt active power filter to improve power quality in transformers"**

**Dear Prof.,**

The authors would like to express their sincere gratitude to the Editors, and the Reviewers for the constructive comments and valuable suggestions to improve the article. The author has revised the paper according to the reviewer’s comments and the editorial directions. The responses to the reviewer’s and editor comments can be found below their respective comments, and the changes can be tracked in the manuscript.

**Response to Reviewer**

**Authors appreciate the valuable comments and expertise in reviewing the paper. Really, the paper quality is improved based on the constructive comments and the corresponding modifications can be tracked in the revised manuscript version.**

**Response to Reviewer#1 Comments**

**Recommendation: Major Revision**

**Comments:**

Reviewer #1: The manuscript presents the research that continues the effort of the authors in the field of harmonics reduction. The idea of using the DQ method based on detecting the positive and negative sequence components is proposed to precisely control the shunt APF was expressed by the first author some years ago (see PhD Dissertation Saad Al-Gahtani). Now the authors continue to apply this idea for power transformers. The system was simulated (using Matlab/Simulink) to evaluate the control system response with and without the proposed APF. The obtained results of the research encourage to continue for other systems also. I suggest to insert in the reference also the paper published in 2023 that was used also: Detection and Classification of Changes in Voltage Magnitude During Various Power Quality Disturbances (2023 4th International Conference for Emerging Technology (INCET) Belgaum, India. May 26-28, 2023) - S Ramana Kumar Joga and all.

**Comment_1:**

Suggestion as follows “I suggest to insert in the reference also the paper published in 2023 that was used also: Detection and Classification of Changes in Voltage Magnitude During Various Power Quality Disturbances (2023 4th International Conference for Emerging Technology (INCET) Belgaum, India. May 26-28, 2023) - S Ramana Kumar Joga and all.”

**Response:**

**Authors appreciate reviewer for his valuable comments and suggestion: As per the suggestion, the suggested research article**

**“Detection and Classification of Changes in Voltage Magnitude During Various Power Quality Disturbances (2023 4th International Conference for Emerging Technology (INCET) Belgaum, India. May 26-28, 2023) - S Ramana Kumar Joga et. Al.”**

**is added as reference, as this paper presents the detail discussion on power quality disturbance classification.**

**Response to Reviewer# 2 Comments**

**Recommendation: Rejection of the work**

**Comments:**

Reviewer #2: The paper is not motivated by a valid scientific question or provides a descriptive analysis of a relevant phenomenon. The paper does not make any innovation over already existing DQ/SRF control theory of SAPF. Just authors made a different presentation of the same on transformer application.

There appear to be limitations in the analysis and data presented that would preclude a meaningful conclusion from being reached, and I do not see a way for these limitations to be readily addressed by revision. Scientific rigour is a fundamental requirement for any research paper and given these concerns regarding the validity of the findings, I recommend strong rejection of this work.

**Response:**

**A developed DQ method based on detecting the positive and negative sequence components is proposed to precisely control the shunt APF for reliable operation of power transformer. This detection technique improves the response time, mitigate the harmonics effecting the operation of transformer and overall power factor. The research article implements the modified/improved DQ method where low pass filter components are eliminated to have fast response in detection and identifying the reference voltage for compensation. This contribution suggests the novelty of the system and can be validated from the results too.**

**Response to Reviewer# 3 Comments**

**Recommendation: Major Revision**

**Comments:**

Reviewer #3: Reviewer’s comments.

**Comment 1:**

1. There is a major drawback in the control methodology as the authors have ignored the zero-sequence components of the current which are essential parts when there are harmonics involved. No discussion is made in this regard.

**Response:**

**Thanks to the reviewer for suggesting an important technical point. As per the suggestion discussion on “why zero sequence components are ignored”, has been added from lines 186 to 193.**

To understand the control method, it is important to know that zero sequence currents and voltages are not present in delta-connected transformers since there is no neutral connection. Therefore, for systems with predominantly delta-connected loads and sources, the consideration of zero sequence components may be unnecessary. Also, system components with no grounding especially in industrial or isolated applications, grounding may be limited or absent. In such cases, zero sequence components may have minimal relevance, as they are primarily associated with ground faults and unbalanced ground currents. Considering this fact, the modified DQ method depends on appearing of the negative sequence components in load current during abnormal conditions.

**Comment 2:**

 2. No significant difference is observed in fig. 9 between the traditional method and the developed method.

**Response:**

**Authors appreciate reviewer for detailly reviewing the topic. It can be observed by zooming the document that there is significant difference in the waveforms, Where more spikes and distortions are available in the traditional DQ compared to developed DQ method. Sometimes, Slight difference will be observed when the system is balanced.**

**Comment 3:**

 3. Issues in the equation numbers as there are only 11 equations while the authors have said equations (1-15)

**Response:**

**Authors appreciate the reviewer for the important comment.**

**Authors accept the typo-error and it is corrected as** “with aid of equations (1-11)”

**Comment 4:**

4. If the control methodology is strictly observed, there is a major flaw, the authors can be asked to justify why they ignored the zero-sequence components, if they have any justification.

**Response:**

**As per the suggestion, the justification for why the zero-sequence components are ignored has been included in the article from lines 186 to 193. ( as presented below)**

To understand the control method, it is important to know that zero sequence currents and voltages are not present in delta-connected transformers since there is no neutral connection. Therefore, for systems with predominantly delta-connected loads and sources, the consideration of zero sequence components may be unnecessary. Also, system components with no grounding especially in industrial or isolated applications, grounding may be limited or absent. In such cases, zero sequence components may have minimal relevance, as they are primarily associated with ground faults and unbalanced ground currents. Considering this fact, the modified DQ method depends on appearing of the negative sequence components in load current during abnormal conditions.

**Comment 5:**

5. Some of the issues are highlighted in yellow in the original doc file.

**Response:**

**As per the suggestion, All the issues highlighted in yellow are addressed and can be tracked.**

**Issues highlighted in yellow**

1) **All the equations were rechecked and necessary corrections are made as per the requirement.**
2) **The representation of transformation ratios is corrected.**
3) **Acknowledgement statement is corrected.**

All the reviewers’ comments have been taken into account in the revised version of the paper which is attached with this response.

**Thank you very much for your cooperation.**

**Sincerely yours.**

*Authors*
