# Supplementary material for: A developed DQ control method for shunt active power filter to improve power quality in transformers
Source: PLoS One. 2024 Jul 18;19(7):e0299635. doi: 10.1371/journal.pone.0299635 (PMC11257387; doi:10.1371/journal.pone.0299635)
Supplement: Attachment — Submitted filename: Response to Reviewers.docx [file pone.0299635.s007.docx]

**Professor Lalit Chandra Saikia, PhD**
Academic Editor,

PLOS ONE

**Subject: Regarding manuscript ID manuscript # PONE-D-23-32919R2**

**Article Title: "** **A developed DQ control method for shunt active power filter to improve power quality in transformers"**

**Dear Prof.,**

The authors would like to express their sincere gratitude to the Editors, and the Reviewers for the constructive comments and valuable suggestions to improve the article. The author has revised the paper according to the reviewer’s comments and the editorial directions. The responses to the reviewer’s and editor comments can be found below their respective comments, and the changes can be tracked in the manuscript.

**Response to Reviewer**

**Authors appreciate the valuable comments and expertise in reviewing the paper. Really, the paper quality is improved based on the constructive comments and the corresponding modifications can be tracked in the revised manuscript version.**

**Additional Editor Comments:**

The paper need minor revision. All the comments made by the reviewer must be addressed and the necessary changes must be made in the manuscript.

Response to the Editor Comments: All the comments by the reviewer are addressed and the necessary changes are made in the manuscript.

**Response to Reviewer#1 Comments**

**Recommendation: Further Revision**

**Comments:**

Reviewer #4:

The paper should be further revised by incorporating the following comments:

**Comment_1:**

The main contributions of this paper should be further summarized and clearly demonstrated. This reviewer suggests the authors exactly mention what is new compared with existing approaches and why the proposed approach is needed to be used instead of the existing methods.

**Response:**

**Authors appreciate reviewer for his valuable comments and suggestion: The main contributions of this article with the comparison from existing approach are discussed in introduction section from line 69 to 84. Also the significance of the new approach is also presented in the same paragraph.**

**Comment 2:**

Introduction should also have subsections: Research gap and motivation; Challenges; Contribution; and paper organization.

**Response:**

**As per the suggestion in introduction sub sections of Research gap and motivation, Contribution, and paper organization are added.**

**are added**

**Comment 3:**

Robustness of the method should also be validated in frequency/time domain.

**Response:**

**Authors appreciate the reviewer for the important comment.**

**Robust of the method is validated in time domain system using the equation (4-11)**

**Comment 4:**

Conclusion should be revised. It should be more focused. Also give future direction of the work.

**Response:**

**As per the suggestion, conclusion is revised and also future work is presented.**

**Comment 5:**

Compare computation burdens of different methods.

**Response:**

**As the low pass filters are eliminated in the proposed control method, the computational burden is reduced and is presented in figure 5 (a and b)**

**Comment 6:**

There are few typo and grammatical errors in the paper.

**Response:**

**As per the suggestion, complete paper is reviewed for grammatical errors and necessary changes were made.**

**Comment 7:**

Literature review should be more strengthen by adding few more papers like <https://doi.org/10.1109/ACCESS.2023.3274732>; <https://doi.org/10.1080/15325008.2023.2258871>

**Response:**

**Authors appreciate reviewer for his valuable comments and suggestion: As per the suggestion, the suggested research articles were added in literature review.**

1) <https://doi.org/10.1109/ACCESS.2023.3274732>

**Power Quality Disturbances Detection and Classification Based on Deep Convolution Auto-Encoder Networks**

Poras Khetarpal, Neelu Nagpal, Mohammed S. Al-Numay, Pierluigi Siano, Yogendra Arya, and Neelam Kassarwani.

2) <https://doi.org/10.1080/15325008.2023.2258871>

**A Dual Fundamental Current Extraction Controller for DSTATCOM in the Distribution System for Power Quality Improvement Under Non-Ideal Source Voltage Condition**

Atma Ram, ,Parsh Ram Sharma, Rajesh Kumar Ahuja &Yogendra Arya

Reviewer #5: Please nominate reviewers when submitting your manuscript to make sure it reaches the reviewers with sufficient background in your topic.

Reviewers are nominated as suggested.

All the reviewers’ comments have been taken into account in the revised version of the paper which is attached with this response.

**Thank you very much for your cooperation.**

**Sincerely yours.**

*Authors*
