# Supplementary material for: A developed DQ control method for shunt active power filter to improve power quality in transformers
Source: PLoS One. 2024 Jul 18;19(7):e0299635. doi: 10.1371/journal.pone.0299635 (PMC11257387; doi:10.1371/journal.pone.0299635)
Supplement: Peer review history

# Peer review: Decision Letter 0

**DOI:** 10.1371/journal.pone.0299635.r001

**Authors:** Lalit Chandra Saikia

17 Oct 2023

PONE-D-23-32919A developed DQ control method for shunt active power filter to improve power quality in transformersPLOS ONE

Dear Dr. elbarbary,

Thank you for submitting your manuscript to PLOS ONE. After careful consideration, we feel that it has merit but does not fully meet PLOS ONE’s publication criteria as it currently stands. Therefore, we invite you to submit a revised version of the manuscript that addresses the points raised during the review process.

==============================

It is required to address all the comments raised by the reviewers. The paper is recommended for Major Review

==============================

Please submit your revised manuscript by Dec 01 2023 11:59PM. If you will need more time than this to complete your revisions, please reply to this message or contact the journal office at plosone@plos.org. When you're ready to submit your revision, log on to https://www.editorialmanager.com/pone/ and select the 'Submissions Needing Revision' folder to locate your manuscript file.

Please include the following items when submitting your revised manuscript:

- A rebuttal letter that responds to each point raised by the academic editor and reviewer(s). You should upload this letter as a separate file labeled 'Response to Reviewers'.
- A marked-up copy of your manuscript that highlights changes made to the original version. You should upload this as a separate file labeled 'Revised Manuscript with Track Changes'.
- An unmarked version of your revised paper without tracked changes. You should upload this as a separate file labeled 'Manuscript'.

If you would like to make changes to your financial disclosure, please include your updated statement in your cover letter. Guidelines for resubmitting your figure files are available below the reviewer comments at the end of this letter.

If applicable, we recommend that you deposit your laboratory protocols in protocols.io to enhance the reproducibility of your results. Protocols.io assigns your protocol its own identifier (DOI) so that it can be cited independently in the future. For instructions see: https://journals.plos.org/plosone/s/submission-guidelines#loc-laboratory-protocols. Additionally, PLOS ONE offers an option for publishing peer-reviewed Lab Protocol articles, which describe protocols hosted on protocols.io. Read more information on sharing protocols at https://plos.org/protocols?utm_medium=editorial-email&utm_source=authorletters&utm_campaign=protocols.

We look forward to receiving your revised manuscript.

Kind regards,

Lalit Chandra Saikia, PhD

Academic Editor

PLOS ONE

Journal requirements:

When submitting your revision, we need you to address these additional requirements.

1. Please ensure that your manuscript meets PLOS ONE's style requirements, including those for file naming. The PLOS ONE style templates can be found at 

https://journals.plos.org/plosone/s/file?id=wjVg/PLOSOne_formatting_sample_main_body.pdf and 

https://journals.plos.org/plosone/s/file?id=ba62/PLOSOne_formatting_sample_title_authors_affiliations.pdf

2. Please note that PLOS ONE has specific guidelines on code sharing for submissions in which author-generated code underpins the findings in the manuscript. In these cases, all author-generated code must be made available without restrictions upon publication of the work. Please review our guidelines at https://journals.plos.org/plosone/s/materials-and-software-sharing#loc-sharing-code and ensure that your code is shared in a way that follows best practice and facilitates reproducibility and reuse.

3. Thank you for stating the following in your Competing Interests section:  

[ok]. 

Please complete your Competing Interests on the online submission form to state any Competing Interests. If you have no competing interests, please state ""The authors have declared that no competing interests exist."", as detailed online in our guide for authors at http://journals.plos.org/plosone/s/submit-now 

 This information should be included in your cover letter; we will change the online submission form on your behalf.

4. In your Data Availability statement, you have not specified where the minimal data set underlying the results described in your manuscript can be found. PLOS defines a study's minimal data set as the underlying data used to reach the conclusions drawn in the manuscript and any additional data required to replicate the reported study findings in their entirety. All PLOS journals require that the minimal data set be made fully available. For more information about our data policy, please see http://journals.plos.org/plosone/s/data-availability.

""Upon re-submitting your revised manuscript, please upload your study’s minimal underlying data set as either Supporting Information files or to a stable, public repository and include the relevant URLs, DOIs, or accession numbers within your revised cover letter. For a list of acceptable repositories, please see http://journals.plos.org/plosone/s/data-availability#loc-recommended-repositories. Any potentially identifying patient information must be fully anonymized.

Important: If there are ethical or legal restrictions to sharing your data publicly, please explain these restrictions in detail. Please see our guidelines for more information on what we consider unacceptable restrictions to publicly sharing data: http://journals.plos.org/plosone/s/data-availability#loc-unacceptable-data-access-restrictions. Note that it is not acceptable for the authors to be the sole named individuals responsible for ensuring data access.

We will update your Data Availability statement to reflect the information you provide in your cover letter.

5. Thank you for stating the following in the Acknowledgments Section of your manuscript: 

[The author extends his appreciation to the Deanship of Scientific Research at King Khalid University for supporting funding this work through General Research Project under grant number (RGP.1/425/44).]

We note that you have provided funding information that is not currently declared in your Funding Statement. However, funding information should not appear in the Acknowledgments section or other areas of your manuscript. We will only publish funding information present in the Funding Statement section of the online submission form. 

Please remove any funding-related text from the manuscript and let us know how you would like to update your Funding Statement. Currently, your Funding Statement reads as follows: 

 [The author(s) received no specific funding for this work.]

Please include your amended statements within your cover letter; we will change the online submission form on your behalf.

[Note: HTML markup is below. Please do not edit.]

Reviewers' comments:

Reviewer's Responses to Questions

**Comments to the Author**

1. Is the manuscript technically sound, and do the data support the conclusions?

The manuscript must describe a technically sound piece of scientific research with data that supports the conclusions. Experiments must have been conducted rigorously, with appropriate controls, replication, and sample sizes. The conclusions must be drawn appropriately based on the data presented. 

Reviewer #1: Yes

Reviewer #2: No

Reviewer #3: Partly

**********

2. Has the statistical analysis been performed appropriately and rigorously? 

Reviewer #1: Yes

Reviewer #2: No

Reviewer #3: No

**********

3. Have the authors made all data underlying the findings in their manuscript fully available?

The PLOS Data policy requires authors to make all data underlying the findings described in their manuscript fully available without restriction, with rare exception (please refer to the Data Availability Statement in the manuscript PDF file). The data should be provided as part of the manuscript or its supporting information, or deposited to a public repository. For example, in addition to summary statistics, the data points behind means, medians and variance measures should be available. If there are restrictions on publicly sharing data—e.g. participant privacy or use of data from a third party—those must be specified.

Reviewer #1: Yes

Reviewer #2: No

Reviewer #3: No

**********

4. Is the manuscript presented in an intelligible fashion and written in standard English?

PLOS ONE does not copyedit accepted manuscripts, so the language in submitted articles must be clear, correct, and unambiguous. Any typographical or grammatical errors should be corrected at revision, so please note any specific errors here.

Reviewer #1: Yes

Reviewer #2: No

Reviewer #3: Yes

**********

5. Review Comments to the Author

Please use the space provided to explain your answers to the questions above. You may also include additional comments for the author, including concerns about dual publication, research ethics, or publication ethics. (Please upload your review as an attachment if it exceeds 20,000 characters)

Reviewer #1: The manuscript presents the research that continues the effort of the authors in the field of harmonics reduction. The idea of using the DQ method based on detecting the positive and negative sequence components is proposed to precisely control the shunt APF was expressed by the first author some years ago (see PhD Dissertation Saad Al-Gahtani). Now the authors continue to apply this idea for power transformers. The system was simulated (using Matlab/Simulink) to evaluate the control system response with and without the proposed APF. The obtained results of the research encourage to continue for other systems also. I suggest to insert in the reference also the paper published in 2023 that was used also: Detection and Classification of Changes in Voltage Magnitude During Various Power Quality Disturbances (2023 4th International Conference for Emerging Technology (INCET) Belgaum, India. May 26-28, 2023) - S Ramana Kumar Joga and all.

Reviewer #2: The paper is not motivated by a valid scientific question or provides a descriptive analysis of a relevant phenomenon. The paper does not make any innovation over already existing DQ/SRF control theory of SAPF. Just authors made a different presentation of the same on transformer application.

There appear to be limitations in the analysis and data presented that would preclude a meaningful conclusion from being reached, and I do not see a way for these limitations to be readily addressed by revision. Scientific rigour is a fundamental requirement for any research paper and given these concerns regarding the validity of the findings, I recommend strong rejection of this work.

Reviewer #3: Reviewer’s comments.

1. There is a major drawback in the control methodology as the authors have ignored the zero-sequence components of the current which are essential parts when there are harmonics involved. No discussion is made in this regard.

2. No significant difference is observed in fig. 9 between the traditional method and the developed method.

3. Issues in the equation numbers as there are only 11 equations while the authors have said equations (1-15)

4. If the control methodology is strictly observed, there is a major flaw,the authors can be asked to justify why they ignored the zero-sequence components, if they have any justification.

5. Some of the issues are highlighted in yellow in the original doc file.

**********

6. PLOS authors have the option to publish the peer review history of their article (what does this mean?). If published, this will include your full peer review and any attached files.

If you choose “no”, your identity will remain anonymous but your review may still be made public.

**Do you want your identity to be public for this peer review?** For information about this choice, including consent withdrawal, please see our Privacy Policy.

Reviewer #1: No

Reviewer #2: No

Reviewer #3: No

**********

[NOTE: If reviewer comments were submitted as an attachment file, they will be attached to this email and accessible via the submission site. Please log into your account, locate the manuscript record, and check for the action link "View Attachments". If this link does not appear, there are no attachment files.]

While revising your submission, please upload your figure files to the Preflight Analysis and Conversion Engine (PACE) digital diagnostic tool, https://pacev2.apexcovantage.com/. PACE helps ensure that figures meet PLOS requirements. To use PACE, you must first register as a user. Registration is free. Then, login and navigate to the UPLOAD tab, where you will find detailed instructions on how to use the tool. If you encounter any issues or have any questions when using PACE, please email PLOS at figures@plos.org. Please note that Supporting Information files do not need this step.

Attachment Submitted filename: Article_Transformer_yellow.docx

Attachment Submitted filename: Reviewers comments.docx

# Author comment: Author response to Decision Letter 0

**DOI:** 10.1371/journal.pone.0299635.r002

7 Nov 2023

Dr. Lalit Chandra Saikia, PhD

Academic Editor

PLOS ONE

Subject: Resubmission of Manuscript ID PONE-D-23-32919,

Dear Dr. Lalit Chandra Saikia,

I hope this letter finds you well. I am writing to resubmit our manuscript entitled "A developed DQ control method for shunt active power filter to improve power quality in transformers" with Manuscript id PONE-D-23-32919 for consideration for publication in PLOS ONE.

Firstly, I would like to extend my sincere gratitude to you and the reviewers for the comprehensive and constructive feedback provided on our initial submission. The comments and suggestions were instrumental in enhancing the quality and clarity of our work.

We have taken great care to address each of the reviewers' comments in our revised manuscript. 

I would like to inform that the authors have declared “No competing interests exist in this research work”.

Enclosed, please find our manuscript with track changes option active, along with a rebuttal letter, and the supplementary materials. We kindly request that you consider our submission for publication in PLOS ONE. We are eager to receive feedback and are committed to making any necessary revisions to ensure that our work meets the high standards of your esteemed journal.

Thank you for considering our submission. We look forward to the possibility of contributing to PLOS ONE and are available to provide any additional information as needed.

Sincerely,

Dr. Elbarbary Z. M. S.

Attachment Submitted filename: Response to Reviewers.docx

# Editor report: Decision Letter 1

**DOI:** 10.1371/journal.pone.0299635.r003

**Authors:** Lalit Chandra Saikia

16 Nov 2023

PONE-D-23-32919R1A developed DQ control method for shunt active power filter to improve power quality in transformersPLOS ONE

Dear Dr. elbarbary,

Thank you for submitting your manuscript to PLOS ONE. After careful consideration, we feel that it has merit but does not fully meet PLOS ONE’s publication criteria as it currently stands. Therefore, we invite you to submit a revised version of the manuscript that addresses the points raised during the review process.

==============================The paper need major revision. Comments of the reviewers must be address.==============================

Please submit your revised manuscript by Dec 31 2023 11:59PM. If you will need more time than this to complete your revisions, please reply to this message or contact the journal office at plosone@plos.org. When you're ready to submit your revision, log on to https://www.editorialmanager.com/pone/ and select the 'Submissions Needing Revision' folder to locate your manuscript file.

Please include the following items when submitting your revised manuscript:

- A rebuttal letter that responds to each point raised by the academic editor and reviewer(s). You should upload this letter as a separate file labeled 'Response to Reviewers'.
- A marked-up copy of your manuscript that highlights changes made to the original version. You should upload this as a separate file labeled 'Revised Manuscript with Track Changes'.
- An unmarked version of your revised paper without tracked changes. You should upload this as a separate file labeled 'Manuscript'.

If you would like to make changes to your financial disclosure, please include your updated statement in your cover letter. Guidelines for resubmitting your figure files are available below the reviewer comments at the end of this letter.

If applicable, we recommend that you deposit your laboratory protocols in protocols.io to enhance the reproducibility of your results. Protocols.io assigns your protocol its own identifier (DOI) so that it can be cited independently in the future. For instructions see: https://journals.plos.org/plosone/s/submission-guidelines#loc-laboratory-protocols. Additionally, PLOS ONE offers an option for publishing peer-reviewed Lab Protocol articles, which describe protocols hosted on protocols.io. Read more information on sharing protocols at https://plos.org/protocols?utm_medium=editorial-email&utm_source=authorletters&utm_campaign=protocols.

We look forward to receiving your revised manuscript.

Kind regards,

Lalit Chandra Saikia, PhD

Academic Editor

PLOS ONE

[Note: HTML markup is below. Please do not edit.]

[NOTE: If reviewer comments were submitted as an attachment file, they will be attached to this email and accessible via the submission site. Please log into your account, locate the manuscript record, and check for the action link "View Attachments". If this link does not appear, there are no attachment files.]

While revising your submission, please upload your figure files to the Preflight Analysis and Conversion Engine (PACE) digital diagnostic tool, https://pacev2.apexcovantage.com/. PACE helps ensure that figures meet PLOS requirements. To use PACE, you must first register as a user. Registration is free. Then, login and navigate to the UPLOAD tab, where you will find detailed instructions on how to use the tool. If you encounter any issues or have any questions when using PACE, please email PLOS at figures@plos.org. Please note that Supporting Information files do not need this step.

# Author comment: Author response to Decision Letter 1

**DOI:** 10.1371/journal.pone.0299635.r004

26 Nov 2023

Professor Lalit Chandra Saikia, PhD

Academic Editor, 

PLOS ONE

Subject: Regarding manuscript ID manuscript # PONE-D-23-32919

Article Title: " A developed DQ control method for shunt active power filter to improve power quality in transformers"

Dear Prof., 

The authors would like to express their sincere gratitude to the Editors, and the Reviewers for the constructive comments and valuable suggestions to improve the article. The author has revised the paper according to the reviewer’s comments and the editorial directions. The responses to the reviewer’s and editor comments can be found below their respective comments, and the changes can be tracked in the manuscript. 

Response to Reviewer

Authors appreciate the valuable comments and expertise in reviewing the paper. Really, the paper quality is improved based on the constructive comments and the corresponding modifications can be tracked in the revised manuscript version.

Response to Reviewer#1 Comments

Recommendation: Major Revision

Comments:

Reviewer #1: The manuscript presents the research that continues the effort of the authors in the field of harmonics reduction. The idea of using the DQ method based on detecting the positive and negative sequence components is proposed to precisely control the shunt APF was expressed by the first author some years ago (see PhD Dissertation Saad Al-Gahtani). Now the authors continue to apply this idea for power transformers. The system was simulated (using Matlab/Simulink) to evaluate the control system response with and without the proposed APF. The obtained results of the research encourage to continue for other systems also. I suggest to insert in the reference also the paper published in 2023 that was used also: Detection and Classification of Changes in Voltage Magnitude During Various Power Quality Disturbances (2023 4th International Conference for Emerging Technology (INCET) Belgaum, India. May 26-28, 2023) - S Ramana Kumar Joga and all.

Comment_1:

Suggestion as follows “I suggest to insert in the reference also the paper published in 2023 that was used also: Detection and Classification of Changes in Voltage Magnitude During Various Power Quality Disturbances (2023 4th International Conference for Emerging Technology (INCET) Belgaum, India. May 26-28, 2023) - S Ramana Kumar Joga and all.”

Response:

Authors appreciate reviewer for his valuable comments and suggestion: As per the suggestion, the suggested research article

“Detection and Classification of Changes in Voltage Magnitude During Various Power Quality Disturbances (2023 4th International Conference for Emerging Technology (INCET) Belgaum, India. May 26-28, 2023) - S Ramana Kumar Joga et. Al.”

 is added as reference, as this paper presents the detail discussion on power quality disturbance classification.

Response to Reviewer# 2 Comments

Recommendation: Rejection of the work

Comments:

Reviewer #2: The paper is not motivated by a valid scientific question or provides a descriptive analysis of a relevant phenomenon. The paper does not make any innovation over already existing DQ/SRF control theory of SAPF. Just authors made a different presentation of the same on transformer application.

There appear to be limitations in the analysis and data presented that would preclude a meaningful conclusion from being reached, and I do not see a way for these limitations to be readily addressed by revision. Scientific rigour is a fundamental requirement for any research paper and given these concerns regarding the validity of the findings, I recommend strong rejection of this work.

Response:

 A developed DQ method based on detecting the positive and negative sequence components is proposed to precisely control the shunt APF for reliable operation of power transformer. This detection technique improves the response time, mitigate the harmonics effecting the operation of transformer and overall power factor. The research article implements the modified/improved DQ method where low pass filter components are eliminated to have fast response in detection and identifying the reference voltage for compensation. This contribution suggests the novelty of the system and can be validated from the results too.

Response to Reviewer# 3 Comments

Recommendation: Major Revision

Comments:

Reviewer #3: Reviewer’s comments.

Comment 1:

1. There is a major drawback in the control methodology as the authors have ignored the zero-sequence components of the current which are essential parts when there are harmonics involved. No discussion is made in this regard.

Response:

Thanks to the reviewer for suggesting an important technical point. As per the suggestion discussion on “why zero sequence components are ignored”, has been added from lines 186 to 193.

To understand the control method, it is important to know that zero sequence currents and voltages are not present in delta-connected transformers since there is no neutral connection. Therefore, for systems with predominantly delta-connected loads and sources, the consideration of zero sequence components may be unnecessary. Also, system components with no grounding especially in industrial or isolated applications, grounding may be limited or absent. In such cases, zero sequence components may have minimal relevance, as they are primarily associated with ground faults and unbalanced ground currents. Considering this fact, the modified DQ method depends on appearing of the negative sequence components in load current during abnormal conditions. 

Comment 2:

 2. No significant difference is observed in fig. 9 between the traditional method and the developed method.

Response:

Authors appreciate reviewer for detailly reviewing the topic. It can be observed by zooming the document that there is significant difference in the waveforms, Where more spikes and distortions are available in the traditional DQ compared to developed DQ method. Sometimes, Slight difference will be observed when the system is balanced.

Comment 3:

 3. Issues in the equation numbers as there are only 11 equations while the authors have said equations (1-15)

Response:

Authors appreciate the reviewer for the important comment.

Authors accept the typo-error and it is corrected as “with aid of equations (1-11)”

Comment 4:

4. If the control methodology is strictly observed, there is a major flaw, the authors can be asked to justify why they ignored the zero-sequence components, if they have any justification.

Response:

As per the suggestion, the justification for why the zero-sequence components are ignored has been included in the article from lines 186 to 193. ( as presented below)

To understand the control method, it is important to know that zero sequence currents and voltages are not present in delta-connected transformers since there is no neutral connection. Therefore, for systems with predominantly delta-connected loads and sources, the consideration of zero sequence components may be unnecessary. Also, system components with no grounding especially in industrial or isolated applications, grounding may be limited or absent. In such cases, zero sequence components may have minimal relevance, as they are primarily associated with ground faults and unbalanced ground currents. Considering this fact, the modified DQ method depends on appearing of the negative sequence components in load current during abnormal conditions. 

Comment 5:

5. Some of the issues are highlighted in yellow in the original doc file.

Response:

As per the suggestion, All the issues highlighted in yellow are addressed and can be tracked.

Issues highlighted in yellow

1) All the equations were rechecked and necessary corrections are made as per the requirement.

2) The representation of transformation ratios is corrected.

3) Acknowledgement statement is corrected.

All the reviewers’ comments have been taken into account in the revised version of the paper which is attached with this response.

Thank you very much for your cooperation.

Sincerely yours.

Authors

Attachment Submitted filename: Response to Reviewers.docx

# Peer review: Decision Letter 2

**DOI:** 10.1371/journal.pone.0299635.r005

**Authors:** Lalit Chandra Saikia

20 Dec 2023

PONE-D-23-32919R2A developed DQ control method for shunt active power filter to improve power quality in transformersPLOS ONE

Dear Dr. elbarbary,

Thank you for submitting your manuscript to PLOS ONE. After careful consideration, we feel that it has merit but does not fully meet PLOS ONE’s publication criteria as it currently stands. Therefore, we invite you to submit a revised version of the manuscript that addresses the points raised during the review process.

 All the comments of the reviewers must be addressed satisfactorily and required changes must be made in the revised manuscript.

Please submit your revised manuscript by Feb 03 2024 11:59PM. If you will need more time than this to complete your revisions, please reply to this message or contact the journal office at plosone@plos.org. When you're ready to submit your revision, log on to https://www.editorialmanager.com/pone/ and select the 'Submissions Needing Revision' folder to locate your manuscript file.

Please include the following items when submitting your revised manuscript:

- A rebuttal letter that responds to each point raised by the academic editor and reviewer(s). You should upload this letter as a separate file labeled 'Response to Reviewers'.
- A marked-up copy of your manuscript that highlights changes made to the original version. You should upload this as a separate file labeled 'Revised Manuscript with Track Changes'.
- An unmarked version of your revised paper without tracked changes. You should upload this as a separate file labeled 'Manuscript'.

If you would like to make changes to your financial disclosure, please include your updated statement in your cover letter. Guidelines for resubmitting your figure files are available below the reviewer comments at the end of this letter.

If applicable, we recommend that you deposit your laboratory protocols in protocols.io to enhance the reproducibility of your results. Protocols.io assigns your protocol its own identifier (DOI) so that it can be cited independently in the future. For instructions see: https://journals.plos.org/plosone/s/submission-guidelines#loc-laboratory-protocols. Additionally, PLOS ONE offers an option for publishing peer-reviewed Lab Protocol articles, which describe protocols hosted on protocols.io. Read more information on sharing protocols at https://plos.org/protocols?utm_medium=editorial-email&utm_source=authorletters&utm_campaign=protocols.

We look forward to receiving your revised manuscript.

Kind regards,

Lalit Chandra Saikia, PhD

Academic Editor

PLOS ONE

Journal Requirements:

Please review your reference list to ensure that it is complete and correct. If you have cited papers that have been retracted, please include the rationale for doing so in the manuscript text, or remove these references and replace them with relevant current references. Any changes to the reference list should be mentioned in the rebuttal letter that accompanies your revised manuscript. If you need to cite a retracted article, indicate the article’s retracted status in the References list and also include a citation and full reference for the retraction notice.

Additional Editor Comments:

The paper need minor revision. All the comments made by the reviewer must be addressed and the necessary changes must be made in the manuscript.

[Note: HTML markup is below. Please do not edit.]

Reviewers' comments:

Reviewer's Responses to Questions

**Comments to the Author**

1. If the authors have adequately addressed your comments raised in a previous round of review and you feel that this manuscript is now acceptable for publication, you may indicate that here to bypass the “Comments to the Author” section, enter your conflict of interest statement in the “Confidential to Editor” section, and submit your "Accept" recommendation.

Reviewer #4: (No Response)

Reviewer #5: All comments have been addressed

**********

2. Is the manuscript technically sound, and do the data support the conclusions?

The manuscript must describe a technically sound piece of scientific research with data that supports the conclusions. Experiments must have been conducted rigorously, with appropriate controls, replication, and sample sizes. The conclusions must be drawn appropriately based on the data presented. 

Reviewer #4: (No Response)

Reviewer #5: Yes

**********

3. Has the statistical analysis been performed appropriately and rigorously? 

Reviewer #4: (No Response)

Reviewer #5: I Don't Know

**********

4. Have the authors made all data underlying the findings in their manuscript fully available?

The PLOS Data policy requires authors to make all data underlying the findings described in their manuscript fully available without restriction, with rare exception (please refer to the Data Availability Statement in the manuscript PDF file). The data should be provided as part of the manuscript or its supporting information, or deposited to a public repository. For example, in addition to summary statistics, the data points behind means, medians and variance measures should be available. If there are restrictions on publicly sharing data—e.g. participant privacy or use of data from a third party—those must be specified.

Reviewer #4: (No Response)

Reviewer #5: Yes

**********

5. Is the manuscript presented in an intelligible fashion and written in standard English?

PLOS ONE does not copyedit accepted manuscripts, so the language in submitted articles must be clear, correct, and unambiguous. Any typographical or grammatical errors should be corrected at revision, so please note any specific errors here.

Reviewer #4: Yes

Reviewer #5: (No Response)

**********

6. Review Comments to the Author

Please use the space provided to explain your answers to the questions above. You may also include additional comments for the author, including concerns about dual publication, research ethics, or publication ethics. (Please upload your review as an attachment if it exceeds 20,000 characters)

Reviewer #4: The paper should be further revised by incorporating the following comments:

1. The main contributions of this paper should be further summarized and clearly demonstrated. This reviewer suggests the authors exactly mention what is new compared with existing approaches and why the proposed approach is needed to be used instead of the existing methods.

2. Introduction should also have subsections: Research gap and motivation; Challenges; Contribution; and paper organization.

3. Robustness of the method should also be validated in frequency/time domain.

4. Conclusion should be revised. It should be more focused. Also give future direction of the work.

5. Compare computation burdens of different methods.

6. There are few typo and grammatical errors in the paper.

7. Literature review should be more strengthen by adding few more papers like https://doi.org/10.1109/ACCESS.2023.3274732; https://doi.org/10.1080/15325008.2023.2258871

Reviewer #5: Please nominate reviewers when submitting your manuscript to make sure it reaches the reviewers with sufficient background in your topic.

**********

7. PLOS authors have the option to publish the peer review history of their article (what does this mean?). If published, this will include your full peer review and any attached files.

If you choose “no”, your identity will remain anonymous but your review may still be made public.

**Do you want your identity to be public for this peer review?** For information about this choice, including consent withdrawal, please see our Privacy Policy.

Reviewer #4: **Yes: **Yogendra Arya

Reviewer #5: No

**********

[NOTE: If reviewer comments were submitted as an attachment file, they will be attached to this email and accessible via the submission site. Please log into your account, locate the manuscript record, and check for the action link "View Attachments". If this link does not appear, there are no attachment files.]

While revising your submission, please upload your figure files to the Preflight Analysis and Conversion Engine (PACE) digital diagnostic tool, https://pacev2.apexcovantage.com/. PACE helps ensure that figures meet PLOS requirements. To use PACE, you must first register as a user. Registration is free. Then, login and navigate to the UPLOAD tab, where you will find detailed instructions on how to use the tool. If you encounter any issues or have any questions when using PACE, please email PLOS at figures@plos.org. Please note that Supporting Information files do not need this step.

# Author comment: Author response to Decision Letter 2

**DOI:** 10.1371/journal.pone.0299635.r006

16 Jan 2024

Professor Lalit Chandra Saikia, PhD

Academic Editor, 

PLOS ONE

Subject: Regarding manuscript ID manuscript # PONE-D-23-32919R2

Article Title: " A developed DQ control method for shunt active power filter to improve power quality in transformers"

Dear Prof., 

The authors would like to express their sincere gratitude to the Editors, and the Reviewers for the constructive comments and valuable suggestions to improve the article. The author has revised the paper according to the reviewer’s comments and the editorial directions. The responses to the reviewer’s and editor comments can be found below their respective comments, and the changes can be tracked in the manuscript

Attachment Submitted filename: Response to Reviewers.docx

# Peer review: Decision Letter 3

**DOI:** 10.1371/journal.pone.0299635.r007

**Authors:** Lalit Chandra Saikia

14 Feb 2024

A developed DQ control method for shunt active power filter to improve power quality in transformers

PONE-D-23-32919R3

Dear Dr. elbarbary,

We’re pleased to inform you that your manuscript has been judged scientifically suitable for publication and will be formally accepted for publication once it meets all outstanding technical requirements.

Within one week, you’ll receive an e-mail detailing the required amendments. When these have been addressed, you’ll receive a formal acceptance letter and your manuscript will be scheduled for publication.

An invoice for payment will follow shortly after the formal acceptance. To ensure an efficient process, please log into Editorial Manager at http://www.editorialmanager.com/pone/, click the 'Update My Information' link at the top of the page, and double check that your user information is up-to-date. If you have any billing related questions, please contact our Author Billing department directly at authorbilling@plos.org.

If your institution or institutions have a press office, please notify them about your upcoming paper to help maximize its impact. If they’ll be preparing press materials, please inform our press team as soon as possible -- no later than 48 hours after receiving the formal acceptance. Your manuscript will remain under strict press embargo until 2 pm Eastern Time on the date of publication. For more information, please contact onepress@plos.org.

Kind regards,

Lalit Chandra Saikia, PhD

Academic Editor

PLOS ONE

Additional Editor Comments (optional):

Reviewers' comments:

Reviewer's Responses to Questions

**Comments to the Author**

1. If the authors have adequately addressed your comments raised in a previous round of review and you feel that this manuscript is now acceptable for publication, you may indicate that here to bypass the “Comments to the Author” section, enter your conflict of interest statement in the “Confidential to Editor” section, and submit your "Accept" recommendation.

Reviewer #4: (No Response)

**********

2. Is the manuscript technically sound, and do the data support the conclusions?

The manuscript must describe a technically sound piece of scientific research with data that supports the conclusions. Experiments must have been conducted rigorously, with appropriate controls, replication, and sample sizes. The conclusions must be drawn appropriately based on the data presented. 

Reviewer #4: (No Response)

**********

3. Has the statistical analysis been performed appropriately and rigorously? 

Reviewer #4: (No Response)

**********

4. Have the authors made all data underlying the findings in their manuscript fully available?

The PLOS Data policy requires authors to make all data underlying the findings described in their manuscript fully available without restriction, with rare exception (please refer to the Data Availability Statement in the manuscript PDF file). The data should be provided as part of the manuscript or its supporting information, or deposited to a public repository. For example, in addition to summary statistics, the data points behind means, medians and variance measures should be available. If there are restrictions on publicly sharing data—e.g. participant privacy or use of data from a third party—those must be specified.

Reviewer #4: (No Response)

**********

5. Is the manuscript presented in an intelligible fashion and written in standard English?

PLOS ONE does not copyedit accepted manuscripts, so the language in submitted articles must be clear, correct, and unambiguous. Any typographical or grammatical errors should be corrected at revision, so please note any specific errors here.

Reviewer #4: (No Response)

**********

6. Review Comments to the Author

Please use the space provided to explain your answers to the questions above. You may also include additional comments for the author, including concerns about dual publication, research ethics, or publication ethics. (Please upload your review as an attachment if it exceeds 20,000 characters)

Reviewer #4: Reviewers have incorporated all comments.

Hence, paper may be considered for acceptance in Plos One Journal.

**********

7. PLOS authors have the option to publish the peer review history of their article (what does this mean?). If published, this will include your full peer review and any attached files.

If you choose “no”, your identity will remain anonymous but your review may still be made public.

**Do you want your identity to be public for this peer review?** For information about this choice, including consent withdrawal, please see our Privacy Policy.

Reviewer #4: **Yes: **Yogendra Arya

**********

# Editor report: Acceptance letter

**DOI:** 10.1371/journal.pone.0299635.r008

**Authors:** Lalit Chandra Saikia

24 May 2024

PONE-D-23-32919R3 

PLOS ONE

Dear Dr. elbarbary, 

I'm pleased to inform you that your manuscript has been deemed suitable for publication in PLOS ONE. Congratulations! Your manuscript is now being handed over to our production team.

At this stage, our production department will prepare your paper for publication. This includes ensuring the following:

* All references, tables, and figures are properly cited

* All relevant supporting information is included in the manuscript submission,

* There are no issues that prevent the paper from being properly typeset

If revisions are needed, the production department will contact you directly to resolve them. If no revisions are needed, you will receive an email when the publication date has been set. At this time, we do not offer pre-publication proofs to authors during production of the accepted work. Please keep in mind that we are working through a large volume of accepted articles, so please give us a few weeks to review your paper and let you know the next and final steps. 

Lastly, if your institution or institutions have a press office, please let them know about your upcoming paper now to help maximize its impact. If they'll be preparing press materials, please inform our press team within the next 48 hours. Your manuscript will remain under strict press embargo until 2 pm Eastern Time on the date of publication. For more information, please contact onepress@plos.org.

If we can help with anything else, please email us at customercare@plos.org.

Thank you for submitting your work to PLOS ONE and supporting open access. 

Kind regards, 

PLOS ONE Editorial Office Staff

on behalf of

Dr. Lalit Chandra Saikia 

Academic Editor

PLOS ONE
